# Track A Basic Science

**DOI:** 10.7448/IAS.15.5.18438

**Published:** 2012-10-22

**Figure 1:**
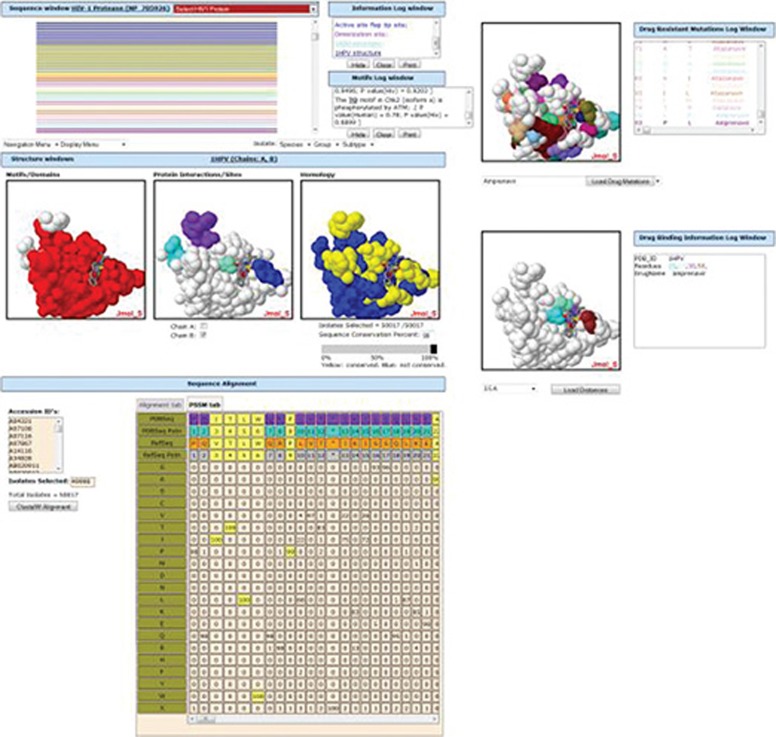
Protease with drug Amprenavir shown [Interactive HIV protein page].

