# Supplementary material for: Track A Basic Science
Source: J Int AIDS Soc. 2012 Oct 22;15(Suppl 3):18438. doi: 10.7448/IAS.15.5.18438 (PMC3499850; doi:10.7448/IAS.15.5.18438)
Supplement: Related texts

# Sub article: THPDA0203: HIVToolbox: an integrated web application for investigating HIV

**Published:** 2012-10-22

**Authors:** D. Sargeant, S. Deverasetty, Y. Luo, A. Villahoz-Baleta, S. Zobrist, V. Rathnayake, J. Russo, M. Muesing, M. Schiller

**Affiliations:** 1 University of Nevada Las Vegas, School of Life Sciences, Las Vegas, United States; 2 University of California Los Angeles, Medical Center, Los Angeles, United States; 3 Aaron Diamond AIDS Research Center, New York, United States; 4 University of Massachusetts, Boston, United States

## Abstract

**Background:**

Many HIV databases and applications focus on a limited domain of HIV knowledge. Since even a “simple” organism like HIV represents a very complex system with many interacting elements, the fractured structure of existing databases and applications likely limits our ability to investigate and understand HIV. To facilitate research, therefore, we have built HIVToolbox, which integrates much of the knowledge about HIV proteins and presents the data in an interactive web application. HIVToolbox allows quick and convenient hypotheses generation, experiment interpretation, and potential new drug structure creation.

**Methods:**

HIVToolbox was built as a standard three-tier J2EE web application, consisting of **1)** an underlying relational MySQL database, **2)** a set of standard Java data access objects that pull data from the database, and **3)** a set of dynamic web pages the user interacts with. HIV-1 data from external sources such as the Protein Data Bank, NCBI, Los Alamos, etc. was collected, curated, and stored in the HIVToolbox database. Additional data, such as homology and position statistics matrices, was generated from existing data. Since version 1, drug binding site and drug resistant mutation data has also been added.

**Results:**

HIVToolbox was used to create several new hypotheses about HIV-1 integrase, including predicting the location of a CK2 phosphorylation site, which was later confirmed by experiment. A new version of HIVToolbox support display of the 3D locations of drug resistant mutations on surface plots of HIV proteins and the drug binding sites for structures of complexes of HIV proteins with drugs.

**Conclusion:**

HIVToolbox is an open-access web application that allows virologists and structural biologists to access detailed information about HIV-1 proteins, such as sequence, structure, functional sites and relationships, homology, drug binding sites, and drug resistant mutations, and to immediately see the relationships between any or all of them. Weblink: [http://hivtoolbox.bio-toolkit.com]

# Sub article: WEAA0103: Production and characterization of human anti-V3 monoclonal antibodies from Indian clade C human immunodeficiency virus type-1 (HIV-1) infected patients

**Published:** 2012-10-22

**Authors:** R. Andrabi, R. Kumar, M. Bala, A. Nair, A. Biswas, N. Wig, P. Kumar, K. Luthra

**Affiliations:** 1 All India Institute of Medical Sciences, Biochemistry, New Delhi, India; 2 Regional STD Teaching Training & Research Centre, Safdarjang Hospital, New Delhi, India; 3 All India Institute of Medical Sciences, Medicine, New Delhi, India; 4 All India Institute of Medical Sciences, Medical Physics Unit, New Delhi, India

## Abstract

**Background:**

Analysis of human monoclonal antibodies (mAbs) developed from HIV-1 infected donors have enormously contributed to the identification of neutralization sensitive epitopes on the HIV-1 envelope glycoprotein. The third variable region (V3) is a crucial target on gp120, primarily due to its involvement in co-receptor (CXCR4 or CCR5) binding and presence of epitopes recognized by broadly neutralizing antibodies.

**Methods:**

Thirty-three HIV-1 seropositive drug naive patients (18 males and 15 females) within the age range of 20-57 years (median=33 years) were recruited in this study for mAb production. The mAbs were selected from EBV transformed cultures with conformationally constrained Cholera-toxin-B containing V3C (V3C-CTB) fusion protein. We tested the mAbs for their binding with HIV-1 derived proteins and peptides by ELISA and for neutralization against HIV-1 viruses by TZM-bl assays.

**Results:**

We isolated three anti-V3 mAbs, 277, 903 and 904 from the cells of different individuals. The ELISA binding revealed a subtype-C and subtype-A specific binding of antibody 277 and 903 while 904 exhibited cross reactivity also with subtype-B V3. Epitope mapping of mAbs with overlapping V3 peptides showed exclusive binding to V3 crown. The antibodies displayed high and low neutralizing activity against 2/5 tier 1 and 1/6 tier 2 viruses respectively. Overall, we observed a resistance of the tier 2 viruses to neutralization by the anti-V3 mAbs, despite exposure of the epitopes recognized by these antibodies on the native viruses, as determined by intact virion binding assay with two representative subtype-C and B viruses (Du156.12 and JRFL).

**Conclusion:**

Our study suggests that the anti-V3 antibodies derived from subtype-C infected Indian patients display neutralization potential against tier 1 viruses. Defining the epitope specificities of these mAbs and further experimental manipulations will be helpful in identification of epitopes, unique to clade C or shared with non-clade C viruses, for immunogen design.

# Sub article: TUPDA0104: Characterization of broadly neutralizing antibodies (bNAbs) to HIV-1 present in a cohort of long term non-progressors (LTNPs)

**Published:** 2012-10-22

**Authors:** N. Gonzalez, K. McKee, E. Yuste, J.R. Mascola, J. Alcamí

**Affiliations:** 1 Instituto de Salud Carlos III, AIDS Immunopathogenesis Unit, Madrid, Spain; 2 Vaccine Research Center, NIH, Bethesda, United States; 3 Hospital Clinic, Barcelona, Spain

## Abstract

**Background:**

One major obstacle to induce bNAbs resides in the high variability of the viral envelope and structural mechanisms hiding crucial epitopes. Besides, maturation of bNAbs against HIV represents a difficult process that can be impaired by the immunodeficiency associated with HIV infection. We have explored the hypothesis that preserved B cell function in LTNPs could result in the production of bNAbs at higher frequency and increased affinity in comparison with HIV progressors.

**Methods:**

Samples (142) from the cohort of LTNPs (median RNA copies/ml: 87, median CD4+: 802 cells/µl) were kindly provided by the HIV BioBank integrated in the Spanish AIDS Research Network (RIS). A control population of 191 untreated patients (median RNA copies/ml: 10,241, median CD4+: 567 cells/µl) from Hospital Clinic, Barcelona, was analyzed. Sera at 1/200 and 1/2000 dilutions were preincubated with Env recombinant viruses harboring a luciferase gene and then added to U87.CD4.CXCR4/CCR5. bNAbs specificities were studied by ELISA using mutated gp120 that abrogates antibody binding, competition ELISA with biotinylated antibodies, neutralization assays with mutated viruses and peptide competition neutralization assays.

**Results:**

The percentage of elite neutralizers was higher in the LTNPs (9.3%) than in the control population (3.7%). Broadly neutralizing sera were screened for the presence of epitope-specific antibodies. CD4 binding site antibodies were detected in several sera. To determine whether these antibodies were responsible for broad neutralization, competition neutralization assays using RSC3 (antigenically resurfaced glycoprotein containing the CD4bs) were performed. RSC3 addition inhibited neutralization mediated by 16.7% of sera in LTNPs and 12.5% sera of the control population. Anti-MPER antibodies were detected in 50% individuals of both populations, including several sera with 4E10-like antibodies. Glycan-dependent HIV-1 NAbs were more abundant in LTNPs (66%) than in control population (37%).

**Conclusion:**

Broad humoral immune responses against HIV-1 were more common among LTNP than a control population of untreated HIV-1-infected donors.

# Sub article: MOAA0202: The majority of freshly sorted SIV-specific CD8^+^ T cells cannot suppress viral replication in SIV-infected macrophages

**Published:** 2012-10-22

**Authors:** L. Vojnov, M. Martins, A. Bean, M. Veloso de Santana, J. Sacha, N. Wilson, M. Bonaldo, R. Galler, M. Stevenson, D. Watkins

**Affiliations:** 1 University of Wisconsin-Madison, Madison, United States; 2 Instituto Oswaldo Cruz-FIOCRUZ, Rio de Janeiro, Brazil; 3 Oregon Health and Science University, Beaverton, United States; 4 Fundação Oswaldo Cruz, Rio de Janeiro, Brazil; 5 University of Miami Miller School of Medicine, Miami, United States

## Abstract

**Background:**

HIV/SIV primarily infect activated CD4^+^ T cells, but can infect macrophages. Because of the relatively small percentage of infected macrophages, the interaction between antigen-specific CD8^+^ T cells and infected macrophages in HIV/SIV infection has been poorly studied. We, therefore, sought to determine whether SIV-specific CD8^+^ T cells could control viral replication in infected macrophages.

**Methods:**

We wanted to ascertain whether *ex vivo* tetramer-sorted SIV-specific CD8^+^ T cells could suppress viral replication in SIVmac239/316e- and SIVsmE660-infected macrophages using a recently developed 48-hour viral suppression assay. We reasoned that freshly sorted CD8^+^ T cells might be more representative of the *in vivo* properties of CD8^+^ T cells than *in vitro* cultured cell lines and clones.

**Results:**

Surprisingly, both *ex vivo* tetramer-sorted SIV-specific CD8^+^ T cells and bulk CD8^+^ T cells that eliminated and suppressed viral replication in SIV-infected CD4^+^ T cells were inefficient at controlling viral replication in SIV-infected macrophages. Our data suggest that macrophages may be an important reservoir for SIV because it may be difficult for SIV-specific CD8^+^ T cells to suppress viral replication in this particular cell type.

**Conclusion:**

It is possible, therefore, that while AIDS virus-infected macrophages only constitute a small percentage of all virus-infected cells, they may be relatively resistant to CD8^+^ T cell-mediated lysis and continue to produce virus over long periods of time. Thus, macrophages could actually be contributing significantly to viral production. Induction of HIV/SIV-specific CD8^+^ T cells capable of killing infected macrophages or preventing establishment of the macrophage reservoir HIV might be critical for controlling viral replication.

# Sub article: MOLBA04: Comparative activity of IgA mediated antibody dependent cell-mediated cytotoxicity (ADCC) in the genital mucosa of HIV seroconverters and highly exposed seronegative women

**Published:** 2012-10-22

**Authors:** M. Aziz, M. Mata, F. Mahmood, H. Durkin, C. Liu, R. Greenblatt, M. Nowicki, E. Golub, K. Anastos, A. French, L. Baum

**Affiliations:** 1 Rush University Medical Center, Chicago, United States; 2 Cook County Health & Hospital Systems, Chicago, United States; 3 SUNY Downstate Medical Center, Brooklyn, United States; 4 Georgetown University, Washington, United States; 5 University of California, San Francisco, United States; 6 University of Southern California Norris Hospital, Los Angeles, United States; 7 Johns Hopkins University, Baltimore, United States; 8 Montefiore Medical Center, Section of Infectious Diseases, Bronx, United States

## Abstract

**Background:**

The potential role of ADCC in prevention of infection is suggested by the RV144 HIV vaccine trial where non-neutralizing antibodies contributed to protection against infection with HIV. Studies from Kenya showed that HIV specific IgA in cervical fluid was present in uninfected sex workers. We aimed to analyze cervicovaginal lavage fluid(CVL) from 2 seroconverters (SC) and 10 highly exposed seronegative women who had unprotected sex with known positive partners.

**Methods:**

A ^51^Cr-release assay with natural killer cells or monocytes as effectors was used to measure IgG or IgA mediated ADCC against clade specific gp120 coupled target cells. We analyzed CVL from ESN at one visit, and SC at intervals from one yr pre-seroconversion (PSC) to 6.5 yr after seroconversion (ASC). We evaluated activity at 4, 10 fold serial dilutions starting with a 1/10 dilution.

**Results:**

Figure 1 (top)shows shows minimal to no activity of IgG mediated ADCC in the CVL of 10 ESN. Figure 1(bottom) shows IgA mediated ADCC in the CVL of the same 10 ESN. 4 patients show significant activity above background activity. At the peak dilution, patient 4 shows 27.3% Specific Release (SR), patient 6 shows 14.5 %SR, patient 8 shows 17.9 %SR and patient 10 shows 17.6 %SR.

Figure 2 and 3 shows no IgA mediated ADCC activity in CVL of 2 seroconverters from PSC to 6.5 years ASC even while IgG activity is present in later visits.

**Conclusion:**

HIV IgA in CVL samples was associated with ADCC and lack of HIV infection in exposed women, indicating that genital HIV IgA may contribute to protection from infection. Further studies need to be done to determine if IgA mediated ADCC antibodies may be protective in ESN.

**Figure 1:** 

**Figure 2:** 

**Figure 3:** 

# Sub article: TUPDA0101: The colocalization potential of HIV-specific CD8+ and CD4+ T cells is mediated by integrin β7 but not CCR6 and regulated by retinoic acid

**Published:** 2012-10-22

**Authors:** V.S. Wacleche, N. Chomont, A. Gosselin, P. Monteiro, M. Goupil, H. Kared, C. Tremblay, N. Bernard, M.-R. Boulassel, J.-P. Routy, P. Ancuta

**Affiliations:** 1 Université de Montréal, Microbiology and Immunology, Montreal, Canada; 2 Centre Hospitalier de l'Université de Montréal- Research Centre, Montreal, Canada; 3 VGTI-Florida, Port St Lucie, United States; 4 INSERM Unit 743, Montreal, Canada; 5 Research Institute of the McGill University Health Centre, Montreal, Canada; 6 Division of Hematology, McGill University Health Centre, Montreal, Canada; 7 Immunodeficiency Service, Montreal Chest Institute, McGill University Health Centre, Montreal, Canada

## Abstract

**Background:**

CD4^+^ T-cells from gut-associated lymphoid tissues (GALT) are major HIV-1 targets. Recruitment of excess effector CD8^+^ T-cells in the proximity of target cells is critical for the control of viral replication. We investigated the colocalization potential of HIV-specific CD8^+^ and CD4^+^ T-cells into the GALT and explored the role of retinoic acid (RA) in regulating this process in a cohort of HIV-infected subjects with slow disease progression (SP).

**Methods:**

Five SP subjects were available for this study: median CD4 counts 670 cells/µl, plasma viral load 3.27 log10 HIV-RNA copies/ml, and 15 years of infection. PBMC were exposed to HIV peptides or CMVpp-65 protein in the presence or absence of all-trans RA (ATRA) or the RA antagonist LE540. The expression of trafficking molecules on antigen specific T-cells was analyzed by flow cytometry using the CFSE assay.

**Results:**

The expression of the gut-homing molecules integrin β7, CCR6, and CXCR3 was identified as a “signature” for HIV-specific but not CMV-specific CD4^+^ T-cells, thus providing a new explanation for their enhanced permissiveness to infection *in vivo*. HIV-specific CD8^+^ T-cells expressed high levels of integrin β7 and CXCR3; however CCR6 was detected at superior levels on HIV-specific CD4^+^ *versus* CD8^+^ T-cells. ATRA upregulated the expression of integrin β7 but not CCR6 on HIV-specific T-cells.

**Conclusion:**

HIV-specific CD8^+^ T-cells may colocalize in excess with CD4^+^ T-cells into the GALT *via* integrin β7 and CXCR3, but not CCR6. Considering our previous findings that CCR6^+^CD4^+^ T-cells are major HIV targets, a limited ability of CD8^+^ T-cells to migrate in the vicinity of CCR6^+^CD4^+^ T-cells may facilitate HIV dissemination at mucosal sites. In addition to other previously described T-cell features (e.g., antiviral properties, poly-functionality, and exhaustion), the colocalization potential of CD4+ and CD8+ T-cells represents a new parameter to consider for predicting the efficacy of anti-HIV responses.

# Sub article: TUPDA0105: Enhanced TCR affinity imparts specificity to reverse transcriptase inhibitor resistance-associated mutations

**Published:** 2012-10-22

**Authors:** J. Jadlowsky, C. Baiduc, M. Richardson, A. Bennett, B. Jakobsen, J.L. Riley

**Affiliations:** 1 University of Pennsylvania, Philadelphia, United States; 2 Adaptimmune, Abingdon, United Kingdom

## Abstract

**Background:**

An attractive approach for restoring CTL activity to HIV-1 infected individuals is the adoptive transfer of autologous CD8 T cells that have been transduced with an HIV-1 specific TCR. Previously, we described an HLA-A2-SL9 specific TCR that when introduced into CD8 T cells could control HIV-1 replication in an *in vitro* suppression assay. Given that the success of HAART is based, in part, on attacking multiple targets, we isolated an additional HLA-A2 restricted, HIV-1 specific TCR targeting the HIV-1_POL_ sequence YQYMDDLYV. This epitope is of great clinical relevance because it lies within the active site of Pol and is a target of many reverse transcriptase inhibitors.

**Methods:**

By surface plasmon resonance, we defined the Kd of this wild type TCR to be 6.7µM and t_1/2_ to be 2.7s. Using phage display, a panel of affinity enhanced A2-YV9 TCRs were obtained with Kd values ranging from 5.1 to 0.3µM.

**Results:**

When introduced into CD8 T cells by lentiviral vectors, the A2 YV9 specific TCRs were highly specific for the wild type epitope. In contrast to what we previously determined for CD8 T cells transduced with the wild type A2-SL9 specific TCR, we observed that the A2-YV9 specific T cells could respond in a polyfunctional manner by simultaneously producing TNF-α, IFN-γ, IL-2, and MIP1-β when presented with a wide range of peptide concentrations. Moreover, affinity enhanced A2-YV9 specific CD8 T cells were able to recognize and respond to several variants of the wild type sequence, including those responsible for resistance to NNRTI and NRTI such as Nevirapine, Didanosine and Efavirenz.

**Conclusion:**

Together, our data suggest that adoptive transfer of these A2-YV9 specific CD8 T cells presents great potential for augmenting available treatments and imparting additional control to HIV-1 infected individuals experiencing drug resistance.

# Sub article: MOAA0201: HIV controllers maintain a population of highly efficient Th1 effector cells in spite of persistently low viral antigenemia

**Published:** 2012-10-22

**Authors:** B. Vingert, D. Benati, O. Lambotte, P. de Truchis, L. Slama, P. Jeannin, S. Perez-Patrigeon, F. Boufassa, W.W. Kwok, F. Lemaître, J.-F. Delfraissy, J. Thèze, L.A. Chakrabarti

**Affiliations:** 1 Institut Pasteur, Unité d'Immunogénétique Cellulaire, Paris, France; 2 INSERM U802, Bicêtre Hospital, Le Kremlin-Bicêtre, France; 3 AP-HP, Department of Infectious and Tropical Diseases, Raymond Poincaré Hospital, Garches, France; 4 AP-HP, Department of Infectious and Tropical Diseases, Tenon Hospital, Paris, France; 5 INSERM U822, Bicêtre Hospital, Le Kremlin-Bicêtre, France; 6 Benaroya Research Institute at Virginia Mason, Seattle, United States; 7 Unité de Dynamiques des Réponses Immunes and INSERM U668, Institut Pasteur, Paris, France

## Abstract

**Background:**

HIV Controllers are rare individuals who spontaneously control HIV replication in the absence of antiretroviral therapy. To identify parameters of the CD4 response that may contribute to viral control, rather than merely reflect a persistently low viremia, we compared the T helper profile in two groups of patients with more than 10 years of viral suppression: HIV Controllers from the ANRS CO18 cohort (n=26) and efficiently treated patients (n=16).

**Methods:**

Cells specific for immunodominant Gag and CMV peptides were evaluated for the production of 10 cytokines and cytotoxicity markers, and were also directly quantified *ex vivo* by MHC class II tetramer staining.

**Results:**

HIV Controller CD4+ T cells were characterized by a higher frequency of IFN-γ production, perforin+/CD107a+ expression, and polyfunctionality in response to Gag peptides. While IL-4, IL-17, and IL-21 production did not differ between groups, treated patients cells produced more IL-10 in response to Gag and CMV peptides, pointing to persistent negative immunoregulation after long-term antiretroviral therapy. Gag293 tetramer-positive cells were detected at high frequency (0.15%) and correlated positively with IFN-γ producing CD4+ T cells in the Controller group (R=0.84; P=0.01). Tetramer-positive cells were fewer in the HAART group (0.04%) and did not correlate with IFN-γ production, supporting the notion of a persistent immune dysfunction in HIV-specific CD4+ T cells of treated patients.

**Conclusion:**

HIV Controllers maintained a population of highly efficient Th1 effectors directed against Gag in spite of a persistently low antigenemia, while patients treated in the long-term showed a loss of CD4 effector functions.

We previously reported that HIV Controllers harbored a unique population of CD4+ T cells expressing high avidity TCRs directed against Gag293. We propose that high avidity drives continuous Th1 effector differentiation in response to low antigen concentrations and explains the persistence of an activated antiviral response in HIV Controllers.

# Sub article: MOAA0204: Natural control of HIV infection is associated with an isotype switched IgG antibody response to HIV core antigens in patients with ‘non-protective’ HLA-B alleles

**Published:** 2012-10-22

**Authors:** M. French, R. Center, K. Wilson, I. Fleyfel, S. Fernandez, A. Kelleher

**Affiliations:** 1 University of Western Australia, School of Pathology and Laboratory Medicine, Perth, Australia; 2 University of Melbourne, Microbiology and Immunology, Melbourne, Australia; 3 National Serology Reference Laboratory, Melbourne, Australia; 4 University of New South Wales, Kirby Institute, Sydney, Australia

## Abstract

**Background:**

Natural control of HIV infection is associated with CD8^+^ T cell responses to HIV core antigens, encoded by *Gag*, restricted by ‘protective’ HLA-B alleles (HLA-B27, -57, -58). Slower progression of HIV infection is associated with antibodies to HIV core proteins but mechanisms are unclear. Antibody responses that have isotype switched to IgG2 may be particularly effective. We have investigated this further.

**Methods:**

Plasma from 32 HIV controllers (HIV RNA<2000 copies/mL, including 14 elite controllers) and 21 ART-naive non-controllers (CD4^+^ T cell count<100/uL) were assayed for IgG1 and IgG2 antibodies to non-recombinant HIV proteins by western blot (WB) assay and to recombinant (r) p55 (Gag) and gp140 (Env) by ELISA. A positive antibody response was defined as a WB band score of 4 or an ELISA optical density >2SD of non-HIV sera for rp55 antibodies. Antibodies to rgp140 were titred. Controllers were HLA typed by sequenced-based typing using genomic DNA.

**Results:**

Controllers had a positive IgG1 or IgG2 antibody response to one or more core protein (p17, p24) on WB more often than non-controllers (75% vs 28.6%, p=0.0016 and 22% vs 0, p=0.034, respectively). Also, 12.5% of controllers but no non-controllers had a positive IgG2 antibody to rp55 (p=0.14). When results of WB assays and ELISA were combined, 34% of controllers but no non-controllers had positive IgG2 antibodies to core antigens (p=0.04). Positive IgG2 antibodies to core antigens were more common in patients without ‘protective’ HLA-B alleles (57%) than patients with these alleles (16.5%) (p=0.026). Positive IgG1 antibodies to *Pol*-encoded proteins were also more common in controllers (p=0.0003) but IgG2 antibodies were not detected. IgG1 and IgG2 antibodies to envelope antigens showed few differences.

**Conclusion:**

An isotype switched IgG antibody response to HIV core antigens is associated with control of HIV infection in patients without ‘protective’ HLA-B alleles.

# Sub article: MOPDA0104: *ZNDR1* gene affects host susceptibility to HIV-1 infection

**Published:** 2012-10-22

**Authors:** P. An, J. Goedert, S. Donfield, S. Buchbinder, G. Kirk, R. Detels, C. Winkler

**Affiliations:** 1 SAIC-Frederick, Inc., NCI Frederick, Frederick, United States; 2 National Cancer Institute, Infections and Immunoepidemology Branch, Division of Cancer Epidemiology and Genetics, Bethesda, United States; 3 Rho Inc, Chapel Hill, United States; 4 San Francisco Department of Public Health, San Francisco, United States; 5 Johns Hopkins University School of Public Health, Baltimore, United States; 6 School of Public Health, University of California, Los Angeles, United States

## Abstract

**Background:**

A recent genome-wide association study (GWAS) of host determinants for HIV-1 disease revealed that single nucleotide polymorphisms (SNPs) near genes *HLA-C* and *ZNRD1* were associated with setpoint HIV-1 viral load and disease progression. ZNRD1 has also been identified as a host protein required by HIV-1 life cycle in a genome-wide functional genomic study.

**Methods:**

We investigated the effects of 13 SNPs in the *ZNRD1* region on HIV-1 infection and progression in five U.S-based treatment-naive HIV-1 longitudinal cohorts consisting of homosexuals, hemophiliacs and injection drug users (IDUs) (n=1028). Allelic frequencies were compared between HIV-1 seronegatives (SN) and seroconverters (SC) with further analysis focusing on high-risk exposed HIV-1-uninfected individuals (HREU) compared to HIV-1 seroconverters (SC). Among HIV- 1 seroconverters, variation in time to clinical AIDS was assessed by haplotype. Electrophoretic mobility shift assay (EMSA) was used to assess the associated SNP's potential to alter DNA-protein interactions.

**Results:**

A haplotype in the *ZNRD1* gene showed significant association with decreased risk of HIV-1 acquisition (OR=0.65, 95% CI 0.47-0.89), independent of the effect of *HLA-C* rs9264942. The tag SNP allele in the *ZNRD1* promoter region causes a loss of nuclear factor binding, as revealed by EMSA. This differential binding was further shown to be cell-specific, occurring in Hela epithelial cells instead of Jurkat T-cells (stimulated or unstimulated), suggesting its regulatory role in mucosal epithelial barriers influence HIV-1 transmission. Indeed, this infection effect was not observed in HIV-1 infected IDUs (OR=0.82, 95% CI, 0.44-1.54). On the other hand, SNPs and haplotypes for *ZNRD1* modestly affect progression rate to AIDS.

**Conclusion:**

This study provided novel evidence supporting a direct role of ZRND1 in modulating HIV and identified a *ZNRD1* allele as a host resistant factor to HIV-1 acquisition. (Funded by NCI HHSN26120080001E)

# Sub article: TUPDB0201: Evidence for T cell immune quiescence in the genital mucosa of HIV exposed seronegative commercial sex workers

**Published:** 2012-10-22

**Authors:** J. Lajoie, J. Juno, A. Burgener, S. Rahman, K. Mogk, C. Wachihi, J. Mwanjewe, F.A. Plummer, J. Kimani, T.B. Ball, K.R. Fowke

**Affiliations:** 1 University of Manitoba, Winnipeg, Canada; 2 University of Manitoba, Medical Microbiology, Winnipeg, Canada; 3 Kenya AIDS Contol Program, Nairobi, Kenya; 4 Public Health Agency of Canada, Winnipeg, Canada; 5 University of Nairobi, Nairobi, Kenya

## Abstract

**Background:**

Understanding the early events during heterosexual HIV transmission at the genital mucosa is necessary to develop a safe and efficacious HIV microbicide or vaccine. A recent workshop highlighted the benefits of studying Highly Exposed Seronegative (HESN) individuals in order to identify and describe correlates of HIV protection. In an HESN cohort of commercial sex workers in Nairobi, Kenya, we have described a state of reduced systemic T cell immune activatio termed Immune Quiescence. However, the extent of Immune Quiescence at the genital mucosal is not known. This study characterized the female genital mucosal profile of cells, cytokines and chemokines involved in immune activation and lymphocyte recruitment among HESN.

**Methods:**

CVL and plasma from commercial sex workers from the Majengo clinic in Nairobi, Kenya (57 HIV- followed for<3 years; 68 HIV infected and 55 HESN followed for >7 years) were analysed for the presence of 22 cytokines/chemokines and five antiproteases previously associated with resistance to HIV infection. Activation of cervico vaginal cells was analysed by multiparametric flow cytometry.

**Results:**

HESN women have a unique pattern of mucosal chemokine/cytokine expression. HESN subjects showed lower expression of MIG, IP-10 and IL-1a as well as higher levels of antiproteases. Among the HESN women there was a distinct chemokine gradient between the blood and genital mucosa relative to control women.

**Conclusion:**

MIG and IP-10 are important regulators of T cell trafficking to the genital mucosa while IL-1a is an indicator of immune activation. The reduced levels of these cytokines/chemokines together with the unique correlations observed with antiprotease expression among HESN women suggest that the Immune Quiescent phenotype extends to the female genital tract. Reducing the number of activated CD4+ T cells in the FGT could limit cellular targets for HIV infection and may be an important component to resisting HIV infection.

# Sub article: WEPDA0105: Highly HIV exposed seronegative (HESN) sex workers from Nairobi, Kenya have altered innate mucosal immune responses at the level of the female genital tract

**Published:** 2012-10-22

**Authors:** A. Burgener, J. Lajoie, C. Wachihi, J. Kimani, K. Fowke, F.A. Plummer, T.B. Ball

**Affiliations:** 1 University of Manitoba, Winnipeg, Canada; 2 University of Nairobi, Nairobi, Kenya; 3 Public Health Agency of Canada, Winnipeg, Canada; 4 Public Health Agency of Canada, National Laboratory for HIV Immunology, Winnipeg, Canada

## Abstract

**Background:**

For 26 years a group of HESN women from Nairobi, Kenya who can be epidemiologically described as relatively resistant to HIV infection have provided clues towards the identification of natural correlates of protection against HIV-1 infection. Studies of these HIV-1-resistant women suggest they posses a unique mucosal environment which includes the overexpression of specific antiproteases and a unique proinflamatory cytokine expression patern. Here we describe how these factors contribute to protection against HIV infection during mucosal transmission.

**Methods:**

Cervical lavage fluid (CVL) from 277 women were collected from 76 HIV-1-resistant, 120 HIV-1 uninfected, and 97 HIV-1 infected women. CVL protein was analyzed both independently by SELDI-TOF MS and as pooled groups by 2D-LC-FTICR MS. Of the more than 350 unique proteins identified 29 proteins were differentially expressed (> 2-fold cutoff) between HIV-1-resistant women and controls. These findings were confirmed by traditional ELISA and quantitative Western Blot (WB) analysis.

**Results:**

The majority of overexpressed proteins were serpins, their breakdown products (p=2.2 x 10^−8^), and other antiproteases, as well as innate factors with known anti-HIV-1 activity. The overexpression of specific serpins and an epithelial-derived antiproteases was confirmed by ELISA and WB (p=0.004, p=0.05, and p=0.02). Underexpressed proteins in HIV-resistant women included inflammatory proteases and immune response factors. Cytokine/chemokine analysis revealed that antiprotease expression correlated with pro-inflammatory cytokines (p<0.0001). However, this was independent of the elevated antiprotease expression observed in HIV-resistant women who in fact expressed reduced levels of certain inflammatory chemokines (p=0.018).

**Conclusion:**

HIV-1-resistant women have elevated acute phase response antiproteases that may regulate inflammation in the female genital tract. Coupled with elevated expression of anti-viral proteins, this may provide a mucosal environment less susceptible to HIV-1. These antiproteases might contribute to a natural protective environment against HIV-1-infection. Understanding this mechanism could aid in microbicide or therapeutic development.

# Sub article: THPDA0103: Low magnitude and frequency of HSV-2-specific interferon gamma-producing CD4^+^ and CD8^+^ T cell responses detected in HIV-1 heterosexual discordant couples

**Published:** 2012-10-22

**Authors:** T.N. Dieye, A. Chentouffi, M. Camara, S. Sarr, M.V. Tran, M. Seydi, G. Dasgupta, M. Fall, G. Daneau, P.A. Diaw, L. Kestens, P.S. Sow, S. Mboup, L. Benmohamed

**Affiliations:** 1 Cheikh Anta Diop University, Dakar, Senegal, Laboratory of Immunology, Department of Bacteriology & Virology, Centre Hospitalier Universitaire Le Dantec, Dakar, Senegal; 2 University of California Irvine, Laboratory of Cellular and Molecular Immunology, The Gavin Herbert Eye Institute and Department of Ophthalmology, Irvine, United States; 3 Cheikh Anta Diop University, Dakar, Senegal, Centre Hospitalier Universitaire de Fann, Dakar, Senegal; 4 Institute of Tropical Medicine, Antwerp, Belgium, Department of Microbiology, Laboratory of Immunology, Antwerp, Belgium

## Abstract

**Background:**

Herpes simplex virus type 2 (HSV-2), the most frequent cause of genital ulcer disease (GUD), has been shown to play a more important role than any other sexually transmitted infections (STIs) in driving HIV prevalence in Africa. In turn, HIV-1 infection leads to more frequent HSV-2 reactivations and shedding. The exact immune mechanisms involved in this virological negative immuno-synergy are unknown. In the present study we sought to assess whether HIV co-infection would affects HSV-specific T cell immunity.

**Methods:**

Nineteen HSV peptides, derived from HSV-2 glycoproteins gB and gD, were used to analyze the frequency and the magnitude of HSV-2-specific IFN-γ-producing CD4^+^ and CD8^+^ T cell responses in 30 HSV-2 seropositive patients and 17 HSV-2 seronegative individuals in a cohort of heterosexual Senegalese HIV-discordant couples, using ELISpot assay. HIV RNA viral load has been run for HIV infected subjects and CD4 count ran for all subjects using a flow cytometry method.

**Results:**

The magnitude and frequency HSV-2-specific T cell responses was compared between 21 HSV-2 co-infected with HIV-1 and 9 HSV-2 mono-infected individuals. A significantly higher magnitude of IFN-γ-producing T cell responses were observed in HSV-2 infected patients compared to seronegative individuals (median, 61 vs. 0 spots/10^6^ PBMC, *P*=0.001). Moreover, twenty-four (80%) out of 30 HSV-2 seropositive patients showed significant HSV-2-specific IFN-γ-producing T cell responses compared with only 6 (35%) out of 17 HSV-2 negative subjects (*P*<0.001). The HSV-2 mono-infected patients showed significantly higher magnitude of HSV-2-specific T cell responses compared to HSV/HIV co-infected patients (median, 140 vs. 42 spots/10^6^ PBMC, *P*=0.024).

**Conclusion:**

Our finding suggest that co-infection with HIV-1 in HSV-2-infected patients might be associated with reduced HSV-2 cellular immune responses. However, the interaction between HIV and HSV-2 appears complex, and precise longitudinal studies will be required to dissect their exact temporal relationship.

# Sub article: TUAA0304: Oral serum-derived bovine immunoglobulin (SBI) administration leads to duodenal gastrointestinal-associated lymphoid tissue (GALT) CD4^+^ T-lymphocyte increases and improved small intestinal absorption function in an 8-week pilot study in patients with HIV-enteropathy

**Published:** 2012-10-22

**Authors:** D.M. Asmuth, Z.-M. Ma, A. Albanese, S. Devaraj, E. Hodzic, J.-C. Garcia, T.H. Knight, N.M. Flynn, S. Mann, T. Yotter, E. Tsuchida, C.J. Miller

**Affiliations:** 1 University of California Davis Medical Center, Sacramento, United States; 2 University of California at Davis, Davis, United States; 3 Center for Comparative Medicine, Davis, United States; 4 Mather Veterans's Administration Hospital, Mather, United States; 5 University of Texas Children's Hospital, Houston, United States; 6 CARES Clinic, Sacramento, United States

## Abstract

**Background:**

HIV-infection leads to GALT CD4^+^ T-cell depletion that persists despite prolonged antiretroviral therapy (ART). SBI is a medical food that neutralizes bacterial antigens and reduces gut inflammation in animal models.

**Methods:**

Subjects on ART with diarrhea and a thorough negative GI-workup received SBI (EnteraHealth, Ankeny,IA,USA) 2.5 grams BID for 8 weeks. 4-hour urine disaccharide gut permeability and absorption test and duodenal biopsies were obtained before and at 8-weeks. Immunohistochemistry for CD3/CD4 was performed on biopsies and flow cytometry was performed on duodenal single-cell suspensions and PBMCs for lymphocyte subsets. Markers of bacterial translocation and cytokine levels were measured in plasma. Stool was collected for 16S rDNA quantification and sequencing. Median values (interquartile ranges) and nonparametric analysis are reported.

**Results:**

All 8 subjects experienced resolution of GI-related symptoms. D-xylose absorption increased in 7/8 subjects and in those with improvement, the absorption levels increased from 31.4 mgs (28.5, 38.8) to 41.5 mgs (33.7, 45.2)(*p*=0.016). Gut permeability was normal before [0.024% (0.0, 0.048)] and after [0.032% (0.0, 0.047)] intervention (normal<0.050%). Median duodenal tissue CD4^+^ T-cell% was unchanged at 16%. Lipopolysaccharide, sCD14, IFN-γ, IL-10, IL-12p70, IL-8, IL-6, and TNF-α were in the normal range before and unchanged after 8-weeks. Absolute lamina propria CD3/CD4 T-cell density increased from 199 cells/mm^2^ (129, 253) to 274 (216, 370)(p=0.062) after 8-weeks of SBI [normal values 836 cells/mm^2^ (474, 1050)]. Previous studies show increases of less than 50 cells/mm^2^ after 9 months of ART. Log_10_ absolute 16S rDNA stool quantification was unchanged between baseline and week-8 [7.125 cp/gm (6.34, 7.5) to 7.415 (6.4, 7.56)].

**Conclusion:**

SBI improved GI absorption and increased GALT CD4^+^ T-lymphocyte density. Further research is needed to demonstrate whether alterations in gut microbiota or inflammatory milieu impact mucosal immunology. Longer studies are needed to examine the mechanisms of SBI in GALT immune reconstitution and improvement in HIV enteropathy.

# Sub article: TUAA0305: Improved intestinal immunity and cytotoxic potential of T cells in interleukin (IL)-21 treated SIV-infected rhesus macaques

**Published:** 2012-10-22

**Authors:** S. Pallikkuth, L. Micci, Z. Ende, R. Iriele, B. Cervasi, J. Else, G. Silvestri, F. Villinger, S. Pahwa, M. Paiardini

**Affiliations:** 1 University of Miami Miller School of Medicine, Miami, United States; 2 Emory University, Yerkes National Primate Research Center, Atlanta, United States

## Abstract

**Background:**

Interleukin (IL)-21 regulates three immunological functions - Th17 cell homeostasis, differentiation of memory B cells and antibody-secreting plasma cells, and long-term maintenance of functional CD8+T-cells - that are compromised in pathogenic HIV and SIV infections. Since IL-21 availability is reduced during infection, we hypothesized that *in vivo* administration of IL-21 might be beneficial for HIV infected humans.

**Methods:**

We infected 12 rhesus macaques with SIV_mac239_ (i.v.), and then treated 6 of them with rhesus rIL-21-IgFc (50mg/kg, s.c., once weekly for 5 weeks) during the early infection (from day 14 to 42 post-infection). Effects of IL-21 on viral load, immune activation, homeostasis of T-cells and their main subsets as well as T-cell cytotoxic potential have been evaluated in blood and mucosa by q-PCR, IHC, and flow cytometry up to 6 months post-infection. Mann-Whitney test and Spearman correlation were used for statistical analyzes.

**Results:**

IL-21 treatment was safe and did not increase plasma viral load or systemic immune activation. Compared to untreated animals, IL-21 treatment resulted in (i) increased expression of Perforin and GrB in total and virus specific CD8+T-cells of various anatomical sites (P<0.05); and (ii) improved mucosal immunity, with higher levels of Th17 CD4+T-cells (P<0.01) during the treatment period. Interestingly, improved Th17 homeostasis was associated with limited proliferation of intestinal CD4+and CD8+T-cells (P<0.05) and reduced plasmatic level of LPS (P<0.01) at 6 months post-infection.

**Conclusion:**

IL-21 treatment during acute SIV infection beneficially impacts on the cytotoxic potential of T-cells and intestinal immunity - including increased homeostasis of Th17 cells, reduced levels of T-cell activation and limited microbial translocation - without undesirable effects on viral load. IL-21 should be further explored as a potential immunomodulator to be used in HIV infection in the context of ART or as part of new HIV vaccine strategies.

# Sub article: WEPDA0103: Induction and maintenance of HIV-1-specific immune responses in exposed seronegative (ESN) women of serodiscordant couples from southern India

**Published:** 2012-10-22

**Authors:** S. Solomon, K.G. Murugavel, R. Vignesh, D. Bella, R. Shoba, S. Poongulali, N. Kumarasamy, A. Landay, S.S. Solomon, C.R. Swathirajan, P. Balakrishnan, S. Cu-Uvin, K.H. Mayer, B.L. Shacklett

**Affiliations:** 1 YRG Centre for AIDS Research and Education (YRG CARE), Chennai, India; 2 Rush University Medical Center, Chicago, United States; 3 Johns Hopkins University School of Medicine, Baltimore, United States; 4 Brown University-The Miriam Hospital, Rhode Island, United States; 5 Harvard Medical School-Beth Israel Deaconess Medical Center/The Fenway Institute, Boston, United States; 6 University of California-Davis, Davis, United States

## Abstract

**Background:**

The majority of new HIV infections are acquired via heterosexual transmission. Certain individuals remain seronegative despite repeated high-risk exposure to HIV. The correlates of protection in exposed seronegative(ESN) individuals remain unclear. The purpose of this study was to determine the breadth and persistence of HIV-specific CTL responses in blood and cervical mucosa of ESN women.

**Methods:**

The ESN cohort included 30 female partners of antiretroviral naïve HIV+ men. ESN women were followed longitudinally for 3-6 months. Controls included sero-concordant couples (n=17) and low-risk seronegative women (n=30). PBMC and mucosal mononuclear cells (MMC) from cervical cytobrush were stimulated with HIV Gag and Env (Clade C) peptide pools; IFN-g production was measured by intracellular cytokine staining. IFN-g production by>0.1% of cells after background subtraction was considered positive. Samples with <400 total events and/or<10 positive events were considered equivocal.

**Results:**

At baseline, PBMC from 2 of 30(7%) ESN and MMC from 3 of 17 (18%) ESN responded to stimulation with HIV antigens. During follow-up, 17 PBMC and 7 MMC samples were analyzed. Of these, PBMC from 2 ESN(12%) and MMC from 1 ESN(14%) responded to Gag and/or Env. In HIV+ women of concordant couples, responses were detected in 50% of PBMC and 40% of MMC. No responses were detected in low-risk controls. Median CD4 and plasma viral loads of male partners of ESN women were 477 cells/µL (range 238-1,090) and 26,300 copies/mL (range 400-750,000); values for male partners in sero-concordant couples were 520(range 132-1,321) and 31,850 (range 400-492,000).

**Conclusion:**

These findings confirm that HIV-specific T-cell responses can be detected in PBMC and MMC from ESN women. These responses could play a protective role; however, they may simply be indicative of antigen exposure. A small percentage of ESN women continued to show positive responses upon longitudinal follow-up, arguing against a protective role for these responses.

# Sub article: TUAA0104: Glut1: establishing a new paradigm for HIV-1 infection by regulating glucose metabolism and activation in CD4+ T cells in HIV-1-positive subjects

**Published:** 2012-10-22

**Authors:** C.S. Palmer, J. Zhou, S. Saleh, L. Lam, A. Hearps, A. Maisa, C. Pereira, S. Lewin, A. Jaworowski, J.M. McCune, S. Crowe

**Affiliations:** 1 Burnet Institute, Virology, Melbourne, Australia; 2 University of New South WalesSchool of Medical Sciences, Sydney, Australia; 3 Monash University, Melbourne, Australia; 4 Alfred Hospital, Melbourne, Australia; 5 Division of Experimental Medicine, University of California, San Francisco, School of Medicine, San Francisco, United States

## Abstract

**Background:**

A characteristic feature of the early phase of mammalian cells to metabolic stress is an increase in the rate of glucose uptake and metabolism. Glucose transporter 1 (Glut1) is the major glucose transporter in T cells and its expression is increased on CD4+T cells during chronic HIV-1 infection in vivo (Palmer et al., Abstract 1, IAS, 2012). We therefore seek to determine the impact of increased Glut1 expression on glucose metabolism in CD4+T cells from HIV-1-infected subjects.

**Methods:**

The cell surface expression of Glut1 and glucose uptake (2-NBDG) was monitored in CD4+T cells from HIV-1 infected treatment naïve or HIV- controls subjects by flow cytometry. Hexokinase and glycolytic activity was measured by the intracellular concentrations of Glucose-6-phosphate (G-6-P) and L-lactate, respectively. Intracellular PTEN, pAkt (T308) and pAkt (S473) levels determined PI3K-mTOR activity. In vitro HIV-1 infection was performed on PBMCs activated with anti-CD3/CD28 microbeads and IL-7 and incubated with the CXCR4-using NL4.3-GFP virus.

**Results:**

Basal glucose uptake, G-6-P, L-lactate, intracellular p-Akt (T308) and p-Akt (S473) were significantly higher in CD4+Glut1+ vs CD4+Glut1- cells. This corresponded with an overall increased glucose uptake and glycolysis and lower levels of PTEN expression in CD4+T cells from HIV-1+subject vs seronegative individuals. Anti-CD3/CD28-induced Glut1 expression on CD4+ T cells was sensitive to specific inhibition of the Class1B PI3K-y and mTORC1 pathways which also blocked HIV-1 infection of CD4+T cells in vitro.

**Conclusion:**

CD4+T cells from HIV-1 infected patients have increased glucose uptake and glycolytic activity mediated at least in part by the PI3k-y-mTORC1 pathway. Increased Glut1 cell surface expression and glycolysis in CD4+T cells may increase their susceptibility to HIV-1 infection and foster their depletion due to hypermetabolism. Approaches to normalize Glut1 expression or glycolysis in CD4+T cells may offer a platform for interventions to slow HIV-1 disease progression.

# Sub article: WEAA0204: HIV-induced CD4 T cell depletion: an innate host defense gone awry?

**Published:** 2012-10-22

**Authors:** G. Doitsh, N. Galloway, K. Monroe, Z. Yang, O. Zepeda, W.C. Greene

**Affiliations:** 1 The J. David Gladstone Institutes, Virology and Immunology, San Francisco, United States

## Abstract

**Background:**

Progressive depletion of CD4 T cells is a hallmark of AIDS yet the underlying mechanism remains poorly understood. In human lymphoid cultures, most of the dying cells correspond to abortively infected resting CD4 T cells (*Cell* 143:789-901,2010). We have now studied how these cells die.

**Methods:**

Human lymphoid aggregated cultures (HLACs) prepared with human tonsil and spleen were used to recapitulate many of the conditions encountered by HIV *in vivo*. CD4 T cell death is prominent in HLAC following viral infection.

**Results:**

>95% human lymphoid CD4 T cells that die in HLAC are abortively, not productively, infected. These nonpermissive resting cells accumulate incomplete cytosolic viral transcripts that trigger an innate immune response resulting in interferon-beta production and activation of caspase-3 and caspase-1. Most cells die as a consequence of caspase-1-mediated pyroptosis, an intensely inflammatory form of programmed cell death where cytoplasmic contents and pro-inflammatory cytokines (IL-1b) are released. Unexpectedly, HIV-induced CD4 T-cell death and release of inflammatory mediators can be blocked by addition of certain oral sulfonylureas like glimepiride that inhibit P2X_7_ ion channels, and are FDA-approved for the treatment of type II diabetes.

**Conclusion:**

1. Abortively infected lymphoid CD4 T cells do not die due to the action of an HIV virulence factor(s), but rather because of host innate immune response launched against viral DNA produced in these cells.

2. This response likely evolved to protect the host from spread of infection, but the involvement of pyroptosis appears to trigger additional rounds of cell recruitment, infection, cell death, and inflammation.

3. CD4 T-cell depletion is blocked by P2X_7_ inhibitors that may interfere with pyroptosis. Such agents might be clinically useful in combination with classical antiretrovirals, particularly in HIV-infected subjects displaying rapid progression of disease or broad drug resistance.

# Sub article: TUPDA0102: Elite controllers show a unique Tryptophan immunosuppressive catabolism

**Published:** 2012-10-22

**Authors:** M. Patel, M.-A. Jenabian, B. Lebouché, M.-J. Brouillette, C. Tremblay, I. Kema, M.-R. Boulassel, J.-P. Routy

**Affiliations:** 1 Chronic Viral Diseases Service of the McGill University Health Centre, Montreal, Canada; 2 Research Institute of the McGill University Health Centre, Montreal, Canada; 3 Department of Psychiatry, Royal Victoria Hospital, McGill University Health Centre, Montreal, Canada; 4 Department of Microbiology and Immunology, Université de Montréal, Montreal, Canada; 5 Department of Laboratory Medicine, University Medical Center, Groningen, Netherlands; 6 Division of Hematology, McGill University Health Centre, Montreal, Canada

## Abstract

**Background:**

Increased Tryptophan (Trp) catabolism into kynurenine (Kyn) and/or 3-hydroxykynurenine (3OH-kyn) by indoleamine 2,3 dioxygenase (IDO), contributes significantly to the persistent immune activation during HIV infection and plays a detrimental role in T cell responses in advanced AIDS. We herein studied Trp catabolism in elite controllers comparing to other well established groups of HIV patients with different disease outcomes.

**Methods:**

Plasma samples from elite controllers (EC) (n=21), ART-naive (n=96), ART-successfully treated (ST) (n=18), and healthy controls (n=51) were collected. All these groups were standardized for nutritional status (albumin levels and body mass index). Levels of Trp, Kyn and 3OH-kyn were measured using isotope dilution tandem mass spectrometry and the markers of Trp catabolism (Kyn/Trp and 3OH-kyn/Trp ratios) were correlated to clinical data.

**Results:**

In ART-naive patients, viral load was positively associated with Kyn, 3OH-kyn levels and the markers of Trp catabolism (p<0.0001). In addition, these patients presented significantly lower Trp levels compared to controls (p=0.0002) and to ST (p=0.042). Accordingly, both Trp metabolites and Kyn/Trp and 3OH-kyn/Trp ratios were more enhanced in ART-naive *vs* ST and controls. EC who spontaneously control viral load, had the same plasma Trp levels as ART-naive (mean=46.15 *vs* 46.6 µmol/L, p=0.874) but lower than controls and ST (54.37 µmol/L, p=0.018 and 52.57 µmol/L, p=0.138 respectively). Interestingly, EC had significantly lowered Trp metabolites such as Kyn and 3OH-kyn levels compared to ART-naive patients (p=0.0005 and p=0.025 respectively). In contrast, EC had similar Kyn and 3OH-kyn levels to Control and ST suggesting a unique Trp metabolism associated with suppressed viral replication.

**Conclusion:**

Elite controllers show a unique regulation of immunosuppressive Tryptophan catabolism. These findings may be relevant for HIV viral control and eradication.

# Sub article: MOAA0205: IL-7 suppresses transcription of the IL-7 receptor alpha (CD127) gene in human CD8 T cells by inducing the expression of a STAT5-dependent transcriptional repressor

**Published:** 2012-10-22

**Authors:** F. Al-Ghazawi, E. Faller, P. Parmar, J. Kakal, P. MacPherson

**Affiliations:** 1 University of Ottawa, BiochemistryMicrobiology and Immunology, Ottawa, Canada; 2 Ottawa Hospital Research InstituteChronic and Infectious Diseases, Ottawa, Canada; 3 Ottawa Hospital, Ottawa, Canada

## Abstract

**Background:**

In view of the role interleukin (IL)-7 plays in T-cell survival, homeostasis and function it is no surprise expression of the IL-7 receptor alpha-chain (CD127) is tightly regulated. We previously showed that expression of CD127 is suppressed on CD8 T-cells in HIV+ patients and that this suppression is mediated by both IL-7 and the HIV Tat protein. IL-7 down-regulates CD127 transcripts and surface protein through two distinct mechanisms. In this study we examine the mechanism by which IL-7 down-regulates the CD127 gene at the level of transcription.

**Methods:**

CD8 T-cells from HIV-negative volunteers were treated with IL-7 (0.1−10 ng/ml) in the presence or absence of various inhibitors. CD127 transcripts were quantified by qPCR. STAT-5 phosphorylation was measured by flow cytometry. Nuclear run-on assays were utilized to measure the rate of CD127 gene transcription. Candidate CD127 repressors were identified using PCR arrays, qPCR, Western and siRNA-mediated gene knock-down assays.

**Results:**

IL-7 attenuates levels of CD127 transcripts in CD8 T-cells in a time- and dose-dependent manner. Both the full-length transcript and the splice-variant encoding the secreted isoform of CD127 are suppressed by IL-7. We show by nuclear run-on assay that IL-7 suppresses the rate of transcription of the CD127 gene and found no evidence that IL-7 affects the stability of CD127 mRNA. Further, the suppression of CD127 transcripts is dependent on JAK kinase activity and phosphorylation of STAT-5 but not STAT-3. Notably, cycloheximide blocked IL-7's ability to down-regulate CD127 transcripts suggesting IL-7 stimulates the *de novo* synthesis of a transcriptional repressor which in turn suppresses CD127 gene transcription. We recently identified several candidate repressors using PCR arrays and are currently examining their involvement in the transcriptional suppression by siRNA-mediated knockout experiments.

**Conclusion:**

Upon binding to its receptor, IL-7 activates the JAK/STAT-5 signaling and induces the expression of a transcriptional repressor which suppresses CD127 gene transcription.

# Sub article: WEAA0205: Suppressor of cytokine signalling (SOCS) proteins are induced by IL-7 and the interaction of SOCS proteins with the IL-7 receptor alpha (CD127) may play a role in regulating CD127 expression in human CD8 T cells

**Published:** 2012-10-22

**Authors:** F. Al-Ghazawi, P. Parmar, E. Faller, S. Sugden, N. Sant, P. MacPherson

**Affiliations:** 1 University of Ottawa, Biochemistry, Microbiology and Immunology, Ottawa, Canada; 2 Ottawa Hospital Research Institute, Chronic and Infectious Diseases, Ottawa, Canada; 3 Ottawa Hospital, Ottawa, Canada

## Abstract

**Background:**

Interleukin (IL)-7 plays essential roles in T-cell development, homeostasis and activation. Disruption of this cytokine pathway likely contributes to HIV-induced immune deficiency. We previously showed that IL-7 and the HIV Tat protein reduce the half-life of the IL-7 receptor alpha-chain (CD127) in human CD8 T-cells but the mechanism directing CD127 to the proteasome is not yet understood. In this study we examined roles of SOCS proteins in regulating CD127 expression.

**Methods:**

CD8 T-cells isolated from healthy HIV-negative volunteers were treated with IL-7 (0.1–10 ng/ml) in the presence or absence of various inhibitors. SOCS1-7 and CIS transcripts were examined by qPCR and protein expression was measured by Western. The interaction of SOCS proteins with CD127 was examined by Co-IP. Surface CD127 protein expression was measured by flow cytometry. Intracellular localization of SOCS and CD127 protein was examined by confocal microscopy.

**Results:**

IL-7 induces the expression of SOCS1-3 and CIS transcripts in CD8 T-cells via the JAK/STAT-5 signaling pathway in a time- and dose-dependent manner with SOCS2 transcripts increasing 300-fold within 3 hours. While induction of SOCS2 and SOCS3 mRNA was transient, SOCS1 and CIS transcripts remained elevated over baseline for at least 48 hours. Western blot analysis confirmed increased protein expression of the induced SOCS genes. Preliminary data on CD8 T-cells isolated from HIV+patients indicate that the IL-7-mediated up-regulation of SOCS transcripts is significantly decreased compared to healthy controls. IL-7 induces rapid phosphorylation and internalization of CD127 followed by proteasomal degradation. By Co-IP we show SOCS proteins induced by IL-7 physically interact with CD127 and study their cellular localization by confocal microscopy. We hypothesize this interaction directs the receptor to the proteasome.

**Conclusion:**

IL-7 induces the expression of SOCS1-3 and CIS genes through the JAK/STAT-5 pathway. Through physical interaction with CD127, SOCS proteins may direct CD127 to the proteasome for degradation.

# Sub article: WEAA0202: Differential SIV infection patterns of lymph node-resident CD4 T cells distinguishes progressive from nonprogressive SIV infection

**Published:** 2012-10-22

**Authors:** C. Vinton, J. Brenchley, B. Tabb, X.P. Hao, V.M. Hirsch, M. Paiardini, J. Lifson, G. Silvestri, J.D. Estes

**Affiliations:** 1 National Institute of Allergy and Infectious Diseases, National Institutes of Health, Program in Barrier Immunity and Repair and Immunopathogenesis Unit, Bethesda, United States; 2 National Institute of Allergy and Infectious Diseases, National Institutes of Health, Laboratory of Molecular Microbiology, Bethesda, United States; 3 National Cancer Institute, National Institutes of Health, AIDS and Cancer Virus Program, Frederick, United States; 4 National Cancer Institute, National Institutes of Health, Pathology and Histotechnology Laboratory, Frederick, United States; 5 Emory University, Atlanta, United States

## Abstract

**Background:**

Nonhuman primate natural hosts for simian immunodeficiency viruses (SIV) develop a non-resolving chronic SIV infection, but do not develop AIDS. While several hypotheses, such as down-modulation of immune activation and differential surface expression of SIV receptors, have been suggested as putative mechanisms to explain the nonprogressive nature of SIV infection in natural hosts, mechanisms underlying high and maintained levels of plasma viremia without apparent loss of target cells in natural hosts remain unclear.

**Methods:**

Here, we have used flow cytometric sorting, quantitative real-time PCR, immunohistochemistry, and in situ hybridization to study viral infectivity and production within subsets of peripheral blood and lymph node-resident CD4^+^ T cells in cohorts of chronically SIVsmm-infected sooty mangabeys and SIVsmE53-infected rhesus macaques.

**Results:**

We find: (1) infection frequencies among PB and LN-resident CD4^+^ T cells in chronically SIVsmE543-infected RM are significantly higher than those in SIVsmm-infected SM; (2) differential virus targeting is observed among anatomical LN niches and among individual CD4^+^ T cell subsets in SIV-infected RM and SM; (3) lymph node resident T_FH_ cells are preferentially SIV-infected in RM, but these cells are not preferentially infected in SM and; (4) infectivity of central and effector memory CD4^+^ T cells is associated with plasma viremia in RM while infectivity of only effector memory CD4^+^ T cell infectivity is associated with plasma viremia in SM.

**Conclusion:**

These data provide mechanistic insights into the maintenance of immunological function among chronically SIV-infected natural hosts for SIV, provide an explanation as to how natural hosts are able to maintain high levels of plasma viremia without apparent loss of target cells, and may lead to novel gene therapy interventions to recapitulate the natural host phenotype to animals that are susceptible to SIV-induced disease.

# Sub article: TUAA0102: Third generation long-read sequencing of HIV-1 transcripts discloses cell type specific and temporal regulation of RNA splicing

**Published:** 2012-10-22

**Authors:** F. Bushman, S. Sherrill-Mix, K. Ocwieja, R. Mukherjee, M. Brown, J. Chin, R. Custers-Allen, P. David, J. Olson, K. Travers, E. Schadt

**Affiliations:** 1 University of Pennsylvania School of Medicine, Department of Microbiology, Philadelphia, United States; 2 Pacific Biosciences, Menlo Park, United States; 3 Raindance Technologies, Lexington, United States

## Abstract

**Background:**

Transcription of the HIV-1 genome yields an initial pre-mRNA which undergoes complex alternative splicing to produce over multiple spliced mRNAs. Analysis of host cell factors important for HIV replication by genome-wide siRNA screens has emphasized that HIV replication is extremely sensitive to the complement of available splicing factors, suggesting that HIV splicing may be an attractive target for therapeutic intervention. Here, we have used single molecule amplification and third generation sequencing to characterize splice patterns for the patient isolate HIV89.6 under different conditions.

**Methods:**

We infected HOS cells or primary T-cells and carried out single molecule PCR in emulsionsto minimize competition among amplicons. We then used Pacific Biosciences single molecule sequencing to analyze message populations.

**Results:**

Primary sequence read lengths averaged 1.6 Kb, and 5% of reads were over 3.8 Kb. Over 100 different messages was documented, more than doubling the previous number. The HIV sequence reads confirmed all of the known major splice junctions and identified new splice junctions, which create new ORFs in the 89.6 isolate. The presence of these new transcripts was confirmed using RT-PCR. The ORFs encode a novel form of Tat with an altered carboxy terminus and a Rev-Nef fusion (dubbed “Ref”) containing the amino terminal helix of Rev and the carboxy terminal portion of Nef. Using this assay, we found that HIV splicing differed between different cell types, differed between different human donors, and changes over time after infection.

**Conclusion:**

These data illustrate how the diversity and promiscuity of splicing in HIV allows the virus to respond to different cellular environments and provides a continuous supply of evolutionary novelty that can potentially serve as a substrate for natural selection.

# Sub article: WEPDA0205: HIV-1 Tat protein up-regulates human cellular miRNAs involved in T cell apoptosis: requirement of Tat second exon

**Published:** 2012-10-22

**Authors:** M. Sánchez del Cojo, M.R. López-Huertas, E. Mateos, M. Coiras, J. Alcamí

**Affiliations:** 1 National Centre of Microbiology, Instituto de Salud Carlos III, AIDS Immunopatology Unit, Majadahonda, Spain

## Abstract

**Background:**

HIV-1 infected cells have evolved strategies to delay apoptosis but the exact mechanism is unknown. One explanation could be the enhancement of Bcl-2 levels. MicroRNAs (miRNAs) are small non-coding RNAs that participate in the innate immune response. Several cellular miRNAs target viral mRNAs, leading to a decreased viral replication, but viruses may also counteract this effect. In the case of HIV-1, Tat has been described as an RNAi suppressor, although this statement remains controversial. To get better insight into this issue and into the effect of Tat in protection to apoptosis, we have analyzed the miRNA expression pattern in Jurkat cells expressing different forms of Tat.

**Methods:**

The miRNA expression profile of Jurkat cells with stable expression of HIV-1 full-length Tat101 (two-exon protein) or Tat72 (exon 1 isoform) was analyzed with a two color-based array of miRNAs (Exiqon). Differences in miRNA expression were then confirmed by qRT-PCR.

**Results:**

Global down-regulation of cellular miRNAs due to the expression of Tat was not observed. Instead, several specific miRNAs were dis-regulated due to the expression of Tat101 or Tat72, although the expression of the second exon granted a higher modification. We confirmed that cellular miR-21 and miR-222 were up-regulated in Jurkat Tat101 cells. miR-21 plays an important role in apoptosis. Since a higher resistance to FasL-mediated apoptosis was observed in Jurkat Tat101, we established a correlation between this protection and the increased levels of Bcl-2 in Jurkat Tat101. Regarding miR-222, it regulates cell cycle progression. In agreement with this, Jurkat Tat101 cells showed less proliferation capacity than control cells.

**Conclusion:**

HIV-1 Tat does not show a general RNAi suppressor activity but it increases specifically several human miRNAs, conferring cells protection to apoptosis. This phenomenon was dependent on the expression of full-length Tat.

# Sub article: TUAA0101: Discovery of novel transcription factors present only in infected cells

**Published:** 2012-10-22

**Authors:** G. Sampey, R. Van Duyne, I. Guendel, E. Gregg, N. Shafagati, M. Tyagi, R. Easely, Z. Klase, F. Romerio, M. Iglesias-Ussel, S. Nekhai, K. Kehn-Hall, F. Kashanchi

**Affiliations:** 1 George Mason University, NCBID, Manassas, United States; 2 George Washington University, Washington, United States; 3 National Institutes of Health, Bethesda, United States; 4 University of Maryland School of Medicine, Baltimore, United States; 5 Howard University, Washington, United States

## Abstract

**Background:**

Throughout the years many labs have discovered important factors that contribute to the transcription of HIV-1. Most of these factors have been well characterized and their significance has been validated. Some of the critical factors involved in Tat activated transcription include RNA Pol II (associated with LTR), chromatin remodeling complexes (i.e. SWI/SNF), acetyltransferases (i.e. CBP, p300, pCAF), NFκB and components of pTEF-b. Surprisingly, almost none of the published literature has focused on finding these factors as complexes from genuine HIV-1 infected cells. Here we show presence of undiscovered complexes unique to HIV-1 infected cells which may serve as targets of inhibition.

**Methods:**

We utilized a combination of HIV infected cell lines and primary latent cells (both T-cells and monocyte/macrophages) to define changes of protein complexes in infected cells. We utilized large quantities of infected cell lysates for size-exclusion chromatography to find novel kinases (i.e. cdk9/T complexes), and chromatin remodeling complexes, among others in presence of high salt (500 mM).

**Results:**

We found that there are novel cdk9/T complexes ranging from 2 MDa to ~300 KDa present only in T-cells that produce virus. Other components of the pTEF-b are also examined and found to be mostly unchanged in infected vs uninfected cells. Other novel complexes present only in infected cells included kinases for the NFkB pathway and SWI/SNF proteins. Many of these proteins are extremely stable and are targets of drug inhibition. Using a small panel of drugs, we find that many of the kinases utilized for transcription of HIV-1 have varying IC50s depending on the size of the complex and their protein partners.

**Conclusion:**

HIV-1 infected cells contain many novel protein complexes that are yet to be discovered. Here we use a simple method of size exclusion to discover novel complexes that could potentially be used as targets of inhibition in therapeutics.

# Sub article: MOAA0102: Myeloid dendritic cells and HIV latency in resting T cells

**Published:** 2012-10-22

**Authors:** N. Kumar, V. Evans, S. Saleh, C.F. Pereira, P. Ellenberg, P.U. Cameron, S.R. Lewin

**Affiliations:** 1 Monash University, Department of Medicine, Melbourne, Australia; 2 Burnet Institute, Melbourne, Australia; 3 Monash University, Monash Micro Imaging, Melbourne, Australia; 4 Alfred Hospital, Infectious Disease Unit, Melbourne, Australia

## Abstract

**Background:**

Latently-infected resting CD4^+^ T-cells are a major barrier to the eradication of HIV infection. These cells are enriched in lymphoid tissue compared to blood. We hypothesized that interactions between dendritic cells (DC) and resting CD4^+^ T-cells are critical for the establishment and maintenance of HIV latency.

**Methods:**

Resting CD4^+^ T-cells labelled with eFluor670 were cultured alone or with syngeneic DC for 24h prior to infection with a CCR5-tropic, EGFP-reporter virus. Non-proliferating (eFluor670^hi^), non-productively-infected (EGFP^-^) CD4^+^ T-cells were sorted on day 5 post-infection. Latent infection was re-activated and amplified by co-culturing sorted cells with mitogen stimulated PBMC.

**Results:**

Infection of resting CD4^+^ T-cells in the presence of myeloid (m)DC significantly increased latent infection of non-proliferating CD4^+^ T-cells compared to infection of T-cells cultured alone (p=0.0005, n=11). Latent infection was not increased in resting CD4^+^ T-cells co-cultured with plasmacytoid DC (n=11) or monocyte-derived-dendritic-cells (n= 2). Co-culture of mDC with memory (CD45RO^+^) CD4^+^ T-cells but not naïve (CD45RO^-^) CD4^+^ T-cells resulted in latency (n=6). eFluor670^hi^EGFP^-^ CD4^+^ T-cells that had been co-cultured with mDC showed a significant increase in the expression of CD69 (p=0.01, n=8) and PD-1 (p=0.007, n=10), but did not express HLA-DR or Ki67. Treatment of the mDC-T-cell co-cultures with blocking antibodies to the chemokines CCL19 and CXCL10 (shown to induce latent infection in resting CD4^+^ T-cells); the chemokine receptor CXCR3; or the adhesion molecule LFA-1 (binds to ICAM) led to no changes in the frequency of latently-infected CD4^+^ T-cells (n=5). When mDC-T-cell contact was prevented using transwells the number of latently-infected CD4^+^ T-cells was reduced (n=3).

**Conclusion:**

mDC play a key role in the establishment and/or maintenance of HIV latency in resting memory CD4^+^ T-cells. Our results suggest this is likely to be mediated through DC-T-cell contact via alternative pathways to ICAM-LFA-1 binding.

# Sub article: TUAA0201: Unique regulatory mechanisms of CNS-derived HIV-1 LTRs associated with latency

**Published:** 2012-10-22

**Authors:** L. Gray, D. Cowley, E. Crespan, C. Welsh, C. Mackenzie, P. Gorry, S. Wesselingh, M. Churchill

**Affiliations:** 1 Burnet Institute, Centre for Virology, Melbourne, Australia; 2 Monash University, Department of Biochemistry and Molecular Biology, Clayton, Australia; 3 Monash University, Department of Medicine, Clayton, Australia; 4 Monash University, Department of Microbiology, Clayton, Australia; 5 Monash University, Department of Immunology, Melbourne, Australia; 6 The University of Melbourne, Department of Microbiology and Immunology, Melbourne, Australia; 7 South Australian Health and Medical Research Institute, Adelaide, Australia

## Abstract

**Background:**

HIV-1 penetrates the central nervous system (CNS) during early infection, establishing a viral reservoir. While macrophages and microglia represent the sites of productive HIV-1 infection, astrocytes undergo a restricted/latent infection. We recently demonstrated that astrocytes are extensively infected and may therefore constitute a significant potential reservoir of HIV-1 within the CNS. Here, we analyzed HIV-1 promoters (LTR) from matched CNS and non-CNS compartments to determine their role in virus restriction within CNS-derived cells. Determining the regulatory mechanisms of CNS-derived LTRs is essential to understanding the CNS as a HIV-1 viral reservoir, and in developing strategies aimed at HIV-1 eradication.

**Methods:**

HIV-1 LTR sequences from a cohort of HIV-1 autopsy subjects consisting of matched CNS- and non-CNS-derived isolates were examined and their activity was determined in T-cells and SVG astrocyte cells. Electrophoretic mobility shift assays (EMSA) were used to analyze transcription factor binding activity within the core and basal promoter regions of the LTR.

**Results:**

CNS-derived LTRs demonstrated restricted basal transcriptional activity in both T-cells and SVG cells, and non-CNS-derived LTRs showed decreased activity in SVG cells. Restricted basal activity mapped to the three Sp binding motifs, previously shown to be essential for both Tat-independent and Tat-dependent activation of the LTR in T-cells. Further repression in astrocytes was observed due to elevated levels of the repressor Sp3 in SVG cells.

**Conclusion:**

The reduced transcriptional activity observed for CNS-derived HIV-1 promoters was found to correlate with a reduction in Sp1 binding, which mapped to mutations within the core Sp binding motif. Transcriptional activity in SVG cells was further regulated by Sp3, which outcompeted Sp1 for Sp-motif binding. These data suggest that CNS-derived viruses have a reduced capacity to initiate viral transcription in astrocytes and highlights that unique transcriptional mechanisms exist within the CNS, ultimately affecting the fate of viral infection and the development of latency.

# Sub article: TUAA0202: Complete transcriptome analysis of latently infected CD4^+^ T cells

**Published:** 2012-10-22

**Authors:** M. Iglesias-Ussel, L. Marchionni, F. Romerio

**Affiliations:** 1 University of Maryland School of Medicine, Institute of Human Virology, Baltimore, United States; 2 Johns Hopkins University, Sidney Kimmel Comprehensive Cancer Center, Baltimore, United States

## Abstract

**Background:**

Latent reservoirs of HIV-1 consist of cells harboring dormant, stably integrated viral genomes that can be reactivated after cell stimulation. Latent HIV-1 evades immune responses and resists anti-retroviral therapy. CD4^+^ T cells are the major reservoir of latent HIV-1. These cells are very rare and lack distinctive markers, which has hindered their characterization.

**Methods:**

We developed an in vitro model suitable to investigate HIV-1 latency in CD4^+^ T cells. In our system CD4^+^ T cells are activated with dendritic cells and antigen, infected in vitro with HIV-1, and then brought back to quiescence through a resting phase in the presence of interleukin-7. During the resting phase, the latently infected cells generated with our system lack expression of activation markers; do not undergo cellular proliferation and do not sustain viral replication. We sorted latently infected and uninfected cells from the same cell culture at the end of the resting phase, and profiled their entire transcriptome.

**Results:**

The results of this microarray analysis revealed profound differences between latently infected and uninfected cells derived from the same culture. First, a number of genes involved in all major cellular metabolic pathways are down-modulated in latently infected cells. Second, several messengers involved in gene expression (including chromatin organization, transcription, translation, post-translational modification, transport and assembly) are also down-regulated in latently infected cells. Third, genes involved in signal transduction, cell activation, cell proliferation and cell cycle progression are down-modulated in latently infected cells. Finally, several genes encoding cell surface molecules are differently expressed in latently infected vs. uninfected cells.

**Conclusion:**

The establishment of HIV-1 latency does not simply entail suppression of HIV-1 expression, and the return to cell quiescence. Rather, HIV-1 appears to exploit and/or promote suppression of all cellular functions, leading to cell quiescence and viral latency. These results may have therapeutic implication for viral eradication.

# Sub article: TUAA0204: Epigenetic modifications of HIV proviral LTRs: potential targets for cure

**Published:** 2012-10-22

**Authors:** S. Weber, H. Burger, K. Kemal, B. Weiser, K. Korn, K. Anastos, W. Doerfler

**Affiliations:** 1 Erlangen University, Institute for Virology, Erlangen, Germany; 2 Wadsworth Center, New York State Dept. of Health, Albany, United States; 3 Albert Einstein College of Medicine/ Montefiore Medical Center, New York, United States

## Abstract

**Background:**

HIV-1 cure remains elusive despite HAART due to the reservoirs of proviral DNA integrated into the human genome. Efforts to cure HIV-1 therefore need to aim at eliminating proviral DNA from cellular reservoirs. The first epigenetic signal identified in virus infected and uninfected cells has been promoter methylation. Compelling evidence confirms that specific promoter methylation can lead to gene silencing. Previous studies have examined HIV-1 epigenetics mostly in vitro.

**Methods:**

We determined methylation patterns in HIV-1 proviral genomes from PBMCs obtained from 21 individuals with a spectrum of disease progression. The CpGs in the long terminal repeats (LTRs) of proviral DNA were investigated by bisulfite sequencing in up to 85 genomic variants per individual. This approach facilitates the study of the full range of CpG methylation and sequence variability of HIV-1 proviruses under conditions of natural selection in human populations.

**Results:**

In patients with advanced disease, the HIV-1 proviruses remained essentially unmethylated in their LTRs. In one long-term nonprogressor, the percentage of methylated proviruses varied from 0–77% at different times after infection. More important and unexpected was the detection of three specific LTR-located CpG dinucleotides that had been selectively mutated to TpAs in>20 out of the 32 samples analyzed. Comparison to 11 HIV-1 LTR sequences in the Los Alamos HIV database demonstrated that mutations in the sites identified by our study occurred more frequently than at other locations, although the mutations were different from TpAs.

**Conclusion:**

These specific CpGs, possibly including their abutting sequences, might indicate weak spots in the proviral genomes whose sacrifice by mutation to TpAs could enhance the HIV-1 potential for long-term proviral survival. These data suggest that the sites of the mutated CpGs occurring at conserved sites may serve as potential targets for therapeutic interventions to eliminate integrated proviruses.

Grants: DFG-DO165/28-1; NIH-UO1-AI35004

# Sub article: TUAA0205: Unique integration patterns: in vitro model of HIV latency

**Published:** 2012-10-22

**Authors:** S. Saleh, D. Vatakis, P. Cameron, A. Harman, A. Cunningham, S. Lewin

**Affiliations:** 1 Monash University, Medicine, Melbourne, Australia; 2 Burnet Institute, Melbourne, Australia; 3 University of California at Los Angeles, Medicine, Los Angeles, United States; 4 Infectious Diseases Unit, Alfred Hospital, Melbourne, Australia; 5 Westmead Millenium Institute, Sydney, Australia

## Abstract

**Background:**

We have demonstrated that HIV latency can be established in resting CD4+ T-cells infected after incubation with the CCR7 ligand, CCL19. The aim of this study was to identify the sites of viral integration in this model and to determine the relationship of integration sites to transcription factor binding sites and cellular gene expression.

**Methods:**

Resting CD4+ T-cells were incubated with either IL2/PHA, CCL19, or in media alone (unactivated). After 2 days, cells were infected with NL4.3 and the presence of integrated DNA was confirmed at day 4. Total cellular DNA was purified and provirus-host junctions cloned and sequenced and mapped on the human genome using the UCSC Genome Browser. Gene expression in each cell type was determined using Illumina microarrays. Comparisons were made between these in vitro conditions and CD4 T-cells from HIV-infected patients on antiretroviral therapy (ART). Gene ontology was analysed using ClueGo.

**Results:**

We identified unique integration sites in infected CCL19-treated (n=247), IL2/PHA (n=432) and unactivated (n=133) T-cells. Integration occurred in transcriptionally active genes and most were involved in cellular housekeeping and cell signalling pathways. Integration sites were a similar distance from CpG islands, CTCF, pol II and histone methylation and acetylation sites. Compared to IL2/PHA-activated and unactivated cells, integration in CCL19-treated cells was further away from regions with high transcriptional activity (including transcriptional start sites (TSS); (p< 0.0001)) and closer to Long Interspersed Nuclear Elements (p< 0.0001), H4K20me3 (p< 0.0001) (a methylation site mapped to heterochromatin) and H4R3me2 (p< 0.0001; involved in priming gene expression). CCL19 treated cells and patient derived cells were similar in some but not all integration site patterns.

**Conclusion:**

HIV integration occurred in transcriptionally active genes in all culture conditions although integration patterns in CCL19-treated latently infected cells were distinct. The significance of unique integration site selection in the setting of latency warrant further investigation.

# Sub article: THAA0104: SIVagm infection of rhesus macaques: a model of functional cure with persistent reservoirs of replication-competent virus

**Published:** 2012-10-22

**Authors:** D. Ma, A. Cillo, C.L. Xu, J. Kristoff, J. Fang, G. Haret-Richter, J. Mellors, I. Pandrea, C. Apetrei

**Affiliations:** 1 University of Pittsburgh, Center for Vaccine Research, Pittsburgh, United States; 2 University of Pittsburgh, Division of Infectious Diseases, Department of Medicine, Pittsburgh, United States

## Abstract

**Background:**

SIVagm infection of rhesus macaques (RMs) provides a model of functional cure: initial high level viremia (10^8^ copies/ml) and massive mucosal CD4^+^ T cell depletion are followed by durable control of SIVagm replication, complete recovery of CD4^+^ T cells, normalization of T-cell activation and seroreversion. The advantage of this model is that the functional cure occurs in all SIVagm-infected RMs. Immune control of SIVagm replication can be temporary reversed by experimental CD8-cell depletion.

**Methods:**

Our objectives were to further characterize the RM/SIVagm model of functional cure by: (i) assessing the level of persistent chronic SIVagm viremia using qPCR with single copy sensitivity (SCA); (ii) determining whether rebounding virus after CD8-cell depletion is replication-competent by inoculation of uninfected RMs; and (iii) characterizing the diversity of rebounding virus using single genome sequencing (SGS).

**Results:**

SCA revealed low level viremia, averaging 20 copies/ml (range 10–30), in RMs 9 month after viremia became undetectable by conventional qPCR. Inoculation of new RMs with plasma collected during virus rebound after CD8 cell depletion resulted in peak viremia of 10^8^–10^9^ SIVagm RNA copies/ml, followed by control of viremia with kinetics similar to that following infection with high titer SIVagm stock virus. SGS of rebound plasma virus after CD8 cell depletion revealed sequence homogeneity consistent with clonality. Rebound virus was genetically similar to unpassaged SIVagm used to infect RMs, suggesting that the viral reservoirs that were the source of the rebounding virus were seeded early after infection.

**Conclusion:**

These findings further validate SIVagm-infected RMs as a model of functional cure of replication-competent retrovirus infection. Deciphering the mechanisms of control may identify new strategies to achieve functional cure. This model is well suited to assess new therapeutic strategies to deplete viral reservoirs without the complexity of multidrug antiretroviral therapy.

# Sub article: MOLBA01: Unintegrated HIV-1 generates an inducible reservoir of replication competent virus in nonproliferating CD4^+^ T cells

**Published:** 2012-10-22

**Authors:** B. Trinité, E. Ohlson, S. Rana, J. Alster, D.N. Levy

**Affiliations:** 1 New York University College of Dentistry, Basic Science, New York, United States

## Abstract

**Background:**

Integration into host cell chromosomal DNA is considered an essential step in the replication of retroviruses, yet HIV-1 replication in vivo or in vitro generates one to two orders of magnitude more copies of unintegrated viral DNA (uDNA) than successfully integrated proviruses (iDNA). These extrachromosomal species are reported to possess limited capacity for gene expression and to be a replicative dead end. Resting CD4^+^ cells are the major targets of early infection following mucosal transmission, and resting memory CD4^+^ T cells constitute the major reservoir of latent infection. The cytokine environment in mucosal and lymphoid compartments facilitates HIV-1 infection of CD4^+^ T cells in the absence of TCR mediated activation.

**Methods:**

We employed a combination of HIV-1 reporter viruses, flow cytometry and quantitative PCR to analyze HIV-1 early and late gene expression and virus production in purified peripheral blood CD4^+^ T cells.

**Results:**

We find that resting CD4^+^ T cells rendered permissive to HIV-1 replication by cytokines IL-2, IL-4, IL-7 or IL-15 provide a reservoir for the persistence of unintegrated HIV-1 DNA. Nonproliferating cells containing uDNA could generate de novo HIV-1 and transmit virus efficiently to uninfected cells, resulting in recombination between viruses. uDNA generated an order of magnitude less virus than integrated proviruses, but cells generating virus from uDNA survived and produced virus longer. Vpr packaged in virions was necessary for initial gene expression from uDNA. Subsequent T cell receptor/coreceptor-mediated activation substantially increased early viral gene expression from uDNA, but an increase in virus production was observed only when activation-induced cell proliferation was inhibited by de novo Vpr generated from the uDNA template. Activation through the T cell receptor or HDAC inhibitors in combination with Prostratin efficiently activated latent uDNA several weeks after infection of resting T cells.

**Conclusion:**

We propose unintegrated HIV-1 as a potential reservoir of inducible virus.

# Sub article: WEPDA0202: Role of UHRF1 in transcriptional regulation and maintenance of HIV-1 latency

**Published:** 2012-10-22

**Authors:** A.F.B. Victoriano, Y. Hibi, N. Tan Gana, K. Asamitsu, T. Okamoto

**Affiliations:** 1 Nagoya City University Graduate School for Medical Sciences, Department of Molecular and Cellular Biology, Nagoya, Japan

## Abstract

**Background:**

A thorough understanding of the molecular mechanisms governing HIV-1 latency is essential in the development of rational therapeutics for the eradication of the virus. Evidence is accumulating that histone methylation regulates HIV latency. The multi-domain protein UHRF1 (ubiquitin-like, containing PHD and RING finger domains 1), a key epigenetic regulator for maintaining DNA methylation patterns, has also been reported to interact with histone 3 lysine 9 methylated histones. This study investigates the role of UHRF1 in the transcriptional silencing of latent HIV-1 provirus.

**Methods:**

293T cells were co-transfected with wild type HIV-1 long terminal repeat (LTR) and either with constructs encoding wild type or mutant forms of human UHRF1, treated with tumor necrosis factor alpha (TNF-α) and the promoter activity was determined by the dual luciferase assay. The presence of UHRF1 at the LTR was assessed by chromatin immunoprecipitation assay using sheared chromatin lysates from latently infected cells, ACH-2 and J-Lat 6.3. Small interfering RNA (siRNA) experiments were conducted using TZM-bl cells, which contain a chromatin-integrated HIV-1 LTR, to confirm the influence of UHRF1 on the HIV-1 LTR.

**Results:**

We observed that UHRF1 inhibited both basal and the induced HIV-1 gene expression by TNF-α. Chromatin immunoprecipitation assay revealed the presence of UHRF1 at the vicinity of the HIV-1 LTR and UHRF1 occupancy was reduced upon activation. Meanwhile, knockdown of UHRF1 expression modestly increased basal LTR activity.

**Conclusion:**

Results suggest that UHRF1 contributes to the transcriptional silencing of latent HIV-1 provirus and further elucidate the underlying molecular mechanisms that maintain latency.

# Sub article: WEPDA0203: Modeling HIV latency using the humanized BLT mouse

**Published:** 2012-10-22

**Authors:** M. Marsden, M. Kovochich, N. Suree, S. Shimizu, R. Mehta, R. Cortado, G. Bristol, D.S. An, J. Zack

**Affiliations:** 1 UCLA, Los Angeles, United States

## Abstract

**Background:**

Replication-competent HIV persists in patients who are treated with highly active antiretroviral therapy (HAART). One significant reservoir of this persistent virus is within rare latently infected CD4+ T cells. However, the infrequent nature of these cells makes them challenging to study directly in infected patients, and clinical attempts to completely eliminate this viral reservoir have not been successful. Therefore improved models for HIV latency and eradication strategies are needed. The humanized bone marrow-liver-thymus (BLT) mouse provides robust multi-lineage immune reconstitution with human cells. When infected with HIV, these mice can also serve as an *in vivo* model for investigating HIV latency.

**Methods:**

BLT mice were infected with HIV and assessed for the presence of latently-infected cells. Cells were stimulated ex vivo with a variety of canonical and novel latency activators. Infected mice were also treated with HAART and then assessed for the presence of activation-inducible virus.

**Results:**

Up to 3% percent of human cells in spleen, peripheral blood, and thymus/liver implants in HIV-infected BLT mice harbored latent HIV. This virus was integrated, activation-inducible, and replication competent. The latently-infected cells were also responsive to stimulation with protein kinase C activators and latency-activating nanoparticles. Furthermore, activation-inducible virus was detectable in HAART-treated mice, although at lower frequencies than in untreated mice.

**Conclusion:**

The humanized BLT mouse provides a versatile system for *ex vivo* and *in vivo* investigation of HIV latency.

# Sub article: WEPDA0204: Maraviroc (MVC) can activate NFkB in resting CD4 T cells of patients infected with R5 or D/M HIV-1

**Published:** 2012-10-22

**Authors:** N. Madrid-Elena, B. Hernandez-Novoa, M. Lamas, L. Garcia-Bermejo, S. Moreno

**Affiliations:** 1 Hospital Ramon y Cajal, Madrid, Spain

## Abstract

**Background:**

In a previous ART intensification study with MVC we detected episomal 2-LTR-DNA in all patients at week 24, while undetectable at baseline (Gutierrez C, et al. *PLoS ONE*, 2011). A residual agonistic effect of MVC on CCR5 receptor through calcium flux has been discarded. Activation of CCR5 intracellular signaling pathways leading to transcription factors activation, including NFkB, could promote HIV-1 transcription in resting CD4 T cells. We aimed to study if MVC could trigger this effect.

**Methods:**

TROPISMVC (NCT01060618) is a clinical trial of 10 days MVC monotherapy in naïve HIV-1-infected patients. Blood samples were drawn at baseline, after 10 days of MVC and 18 days after MVC withdrawal (day 28). PBMCs were isolated from nine patients, bearing CCR5 (n=6) and D/M (n=3) tropic viruses. Resting CD4+ T cells were separated by MACS^®^ Technology and aliquots of 5 million cells were frozen. Nuclear proteins were obtained using the Actif Motif Nuclear Extract Kit. NFkB activation was detected by ELISA plates coated with oligonucleotides mimicking the specific consensus binding sites (TransAM^TM^ NFkB family, Actif Motif), following the manufacturer's instructions. NFkB activity was estimated measuring target genes' expression by real-time PCR of the extracted RNA.

**Results:**

NFkB activity was detected in 4/6 patients with R5 tropic viruses and in 2/3 patients with D/M tropic viruses; results expressed in fold change (FC) compared to baseline according to HIV-1 tropism. The presence of MVC increased NFkB activity consistently, as summarized in the following table. Upregulation of at least one NFkB targeted gene was observed in all but one cases with available RNA sample.

**Conclusion:**

MVC can activate NFkB, and the expression of targeted genes, in resting CD4 T cells from HIV-infected patients regardless of the viral tropism. Through this pathway, MVC could trigger HIV-1 transcription in resting cells thus accelerating the decay of the HIV-1 cell reservoir.

**Table:** 

	Resting CD4 Tcell									
	
	NF-κB(FC)		IFN-γ(FC)		IL-6, (FC)		IL-10 (FC)		TNF-α(FC)	
					
Pattern (Tropism)	Day 10 (on MVC)	Day 28 (off MVC)	Day 10 (on MVC)	Day 28 (off MVC)	Day 10 (on MVC)	Day 28 (on MVC)	Day 10 (on MVC)	Day 28 (off MVC)	Day 10 (on MVC)	Day 28 (on MVC)
27 (R5)	7.6	10.4	226	164	21.4	5	221	103.8	41	195
28 (R5)	4.6	5.3	<2	<2	<2	<2	<2	<2	<2	<2
35 (RS)	4.6	<2	<2	2	<2	4.2	<2	3.1	<2	<2
29 (R5)	17.6	10.1	NA	NA	NA	NA	NA	NA	NA	NA
50 (D/W)	9.1	10.5	<2	11.2	4.1	4.1	<2	<2	25.3	<2
57 (D/M)	2.6	10.8	MA	MA	<2	<2	NA	MA	446.7	38.8

NFkB activity.

# Sub article: THPDA0205: CXCR4-tropism is associated with the preferential establishment of an HIV-reservoir in naïve CD4+ T cells among HIV-positive Ugandan children receiving antiretroviral therapy

**Published:** 2012-10-22

**Authors:** T. Ruel, J. Achan, W. Huang, H. Cao, P. Li, L.A. Eller, M. Killian, E. Sinclair, E. Charlebois, D.V. Havlir, J. Wong

**Affiliations:** 1 University of Calfornia at San Francisco, Department of Pediatrics, San Francisco, United States; 2 Makerere University College of Health Sciences, Department of Paediatrics, Kampala, Uganda; 3 Monogram Biosciences, South San Francisco, United States; 4 University of Calfornia at San Francisco, Department of Medicine, San Francisco, United States; 5 US Military HIV Research Program, Bethesda, United States

## Abstract

**Background:**

Children have large populations of naive CD4+ T-cells that characteristically express high levels of CXCR4 and low levels of CCR5, compared to memory CD4+ T-cells. We hypothesized that HIV+ Ugandan children infected with CXCR4-tropic virus would exhibit larger HIV-DNA reservoirs in naïve CD4+ T-cells, compared to children infected with CCR5-tropic (R5) virus.

**Methods:**

Cryopreserved PBMC from a convenience sample of 12 HIV+ Ugandan children receiving antiretroviral therapy (ART) with undetectable plasma HIV-RNA (< 400 copies/ml, Amplicor, Roche) were sorted into naïve (CD27+CD45RA+) and memory (CD27-CD45+ and CD45-CD27±) CD3+CD4+ T-cells. HIV-DNA levels were determined using a Taqman assay targeting *gag*, normalized to cellular-DNA content (tert, ABI). Co-receptor tropism was determined using a commercial phenotypic assay (Trophile, Monogram). We calculated 1) the ratio of the prevalence of infection (copies per 10^6^ cells) in naïve to the prevalence in memory CD4+ T-cells and 2) the proportion of the total peripheral CD4+ T-cell HIV-reservoir that is contained in naïve CD4+ T-cells, and compared them between children with R5- and dual/mixed(CXCR4/CCR5, DM)-tropic virus using non-parametric statistics.

**Results:**

Median age was 4.9 (interquartile range 3.5-8.1) years, CD4+T-cell number 743 cells/ul (565-1089), CD4+ T-cell percentage 25 (21-29), and ART duration 95 days (95-147), with 6 subjects each with HIV-envelope-subtypes A and D. R5 virus was identified in 8 and DM virus in 4 children.

**Conclusion:**

In ART-treated adults, the vast majority of persistently infected CD4+ T-cells are memory cells. By contrast, we found that a significant proportion of the reservoir resides in the naïve CD4+ T-cells among Ugandan HIV+ ART-treated children. Infection with DM virus was associated with preferential naïve T-cell infection. In developing strategies to eradicate HIV, it will be important to take into account the high levels of naïve T-cell infection in children, particularly among those with DM virus.

**Table:** 

	HIV Copies/10^6 Naïve CD4+T-Cells	HIV Copies/10^6 Memory CD4+T-cells	Ratio of infection prevalence	HIV+Naive CD4+T-cells/Total HIV+CD4+T-cells
R5	2,962 (129–10,668)	9,931 (1037–11,808)	0.7 (0.2–4.3)	53% (25%–80%)
DM	14,733 (1,344–135,120)	930 (151–29,702)	8.9 (6.6–13.0)	92% (88%–95%)
P-value§	0.31	0.61	0.04	0.07

* Comparing R5 to DM with Kruskal-Wallis Test.

HIV-Reservoir by CCR5/DM Tropism.

# Sub article: THAA0101: Long-term reduction in peripheral blood HIV-1 reservoirs following reduced-intensity conditioning allogeneic stem cell transplantation in two HIV-positive individuals

**Published:** 2012-10-22

**Authors:** T.J. Henrich, G. Sciaranghella, J.Z. Li, S. Gallien, V. Ho, A.S. Lacasce, D.R. Kuritzkes

**Affiliations:** 1 Brigham and Women's Hospital, Boston, United States; 2 Harvard Medical School, Boston, United States; 3 Ragon Institute of MGH, MIT and Harvard, Boston, United States; 4 Hopital Saint-Louis, Paris, France; 5 Dana-Farber Cancer Institute, Boston, United States

## Abstract

**Background:**

Functional HIV-1 cure has been described in the setting of myeloablative allogeneic stem cell transplant (alloSCT) with *ccr5▵32/ ccr5▵32* donor cells, but the effects of alloSCT on viral reservoirs are largely unknown. We studied the longitudinal effects of reduced-intensity conditioning (RIC) alloSCT on HIV-1 peripheral blood reservoirs in two infected male patients with hematologic malignancies who previously underwent autologous SCT.

**Methods:**

Analysis of peripheral HIV-1 reservoirs was performed on banked samples (1 pre- and 3 post-RIC-AlloSCT) for both patients, including: 1) quantification of HIV-1 DNA from peripheral blood mononuclear cells (PBMCs), 2) quantification of 2-LTR circles from PBMC episomal DNA, 3) full-length envelope amplification and phenotypic coreceptor usage prediction from proviral DNA, 4) quantification of plasma viremia by a single-copy assay, 5) flow cytometric characterization of lymphocyte subsets and coreceptor expression, and 6) CCR5 genotyping.

**Results:**

No HIV-1 DNA was detected 8 to 17 months after alloSCT in PBMC from both patients despite presence of modest levels of total PBMC-associated HIV-1 DNA prior to and 2-3 months after SCT (87–271 copies/10^6^ PBMCs). 2-LTR circles were not detected at any time-point despite excellent recovery of episomal mitochondrial DNA. Both patients were heterozygous for *ccr5▵3*2 mutation prior to transplant; a transient reduction in CXCR4 expression was observed following transplant. Pseudoviruses incorporating envelopes from early time-points used predominately CCR5 for entry. Both patients remained virologically suppressed on ART, but were either started on prednisone or continued on tacrolimus/sirolimus immunosuppressive therapy for chronic graft-versus-host disease (GVHD) near the time of loss of HIV-1 reservoir detection.

**Conclusion:**

PBMC HIV-1 DNA became undetectable 8 months after RIC-alloSCT. This finding may be due to a dilutional effect of donor cell engraftment in the setting of protective ART, the additive effect of cytotoxic therapies, and/or GVHD. Confirmation of results by sampling large-volume blood collections and other tissue compartments is warranted.

**Table:** 

Patient	Sample Collection Time (months from allogeneic SCT)	Total HIV-1 DNA (copies/million PBMCs)	2-LTR HIV-1 DNA (copies/million PBMCs)	Plasma HIV-1 RNA (copies/ml)
**A**	−0.5 (pre-SCT)	**144**	ND	<3
**A**	2	**87**	ND	<3
**A**	8	ND	ND	<3
**A**	14	ND	ND	<3
**B**	−0.2 (pre-SCT)	**96**	ND	<3
**B**	3	**281**	ND	<3
**B**	9	ND	ND	<3
**B**	17	ND	ND	<3

ND=Target Not Detected

Quantification of Peripheral Blood HIV Reservoirs.

# Sub article: MOLBA02: Evaluation of treatment with the histone deacetylase inhibitor vorinostat (suberoylanilide hydroxamic acid; SAHA) in antiretroviral drug treated, SIVmac239-infected rhesus macaques

**Published:** 2012-10-22

**Authors:** J. Lifson, G. Del Prete, R. Kiser, C.M. Trubey, J. Smedley, V. Coalter, K. Oswald, R. Shoemaker, R. Fast, Y. Li, A. Lara, A. Wiles, R. Wiles, R. Macallister, R. Sanchez, J. Wai, C. Tan, B. Keele, J. Estes, M. Piatakjr, D. Hazuda

**Affiliations:** 1 SAIC Frederick, Inc., Frederick Naitonal Laboratory for Cancer Research, AIDS and Cancer Virus Program, Frederick, MD, United States; 2 SAIC-Frederick, Inc., Frederick National Laboratory for Cancer Research, Laboratory Animal Sciences Program, Frederick, MD, United States; 3 Merck Research Laboratories, West Point, PA, United States

## Abstract

**Background:**

Nonhuman primate (NHP) models are needed for evaluation of proposed but unproven, and potentially dangerous strategies targeting residual virus and latent reservoirs in AIDS virus-infected subjects receiving suppressive antiretroviral drug treatment (ART), but such models have proven challenging to develop.

**Methods:**

We treated a cohort of 6 Indian rhesus macaques with a novel three class (NRTI, PI, IN-STI) six drug (PMPA/FTC/DRV-RTV/L-870812/L-870564) ART regimen beginning at 4 weeks post-infection with SIVmac239. Peripheral blood CD4+ T cells from ART-treated animals with suppressed viremia were evaluated ex vivo for responses to SAHA, including changes in histone acetylation patterns and induction of expression of SIV. Beginning approximately 26 weeks post infection, animals received four 21 day courses of daily treatment with SAHA, with each course of SAHA separated by an approximately 3 week interval, with continuous ART throughout and longitudinal sampling of blood and lymph nodes for immunological, virological, and pharmacodynamic evaluations. Animals were euthanized and necropsied after the final SAHA dose, while still on ART, and tissues studied virologically.

**Results:**

The ART regimen was feasible, safe and well tolerated over one year of treatment and allowed suppression of plasma viremia to<30 copy Eq/mL. Ex vivo SAHA treatment of CD4+ T cells from ART-suppressed macaques increased histone acetylation and induced SIV expression. SAHA treatment of macaques was safe and well tolerated, and induced measurable in vivo changes in histone acetylation in CD4+ T cells but did not reproducibly impact plasma viremia. Analysis of cell associated viral DNA and RNA levels from blood and tissues is in progress and will be presented.

**Conclusion:**

This study demonstrates the feasibility of developing and applying NHP models for studying AIDS virus reservoirs and eradication strategies, along with the in vivo safety of SAHA treatment at pharmacologically active doses.

# Sub article: THPDA0201: The cure of the 'Berlin patient': why did pre-existing X4-variants not emergence after allogeneic CCR5-Δ32 SCT?

**Published:** 2012-10-22

**Authors:** J. Symons, S. Deeks, G. Hutter, A. Wensing, J. Martin, P. van Ham, L. Vandekerckhove, M. Nijhuis

**Affiliations:** 1 University Medical Center Utrecht, Department of Virology, Utrecht, Netherlands; 2 University of California, Department of Medicine, San Francisco, United States; 3 Heidelberg University, Institute of Transfusion Medicine and Immunology, Mannheim, Germany; 4 Ghent University Hospital, Department of Internal Medicine, Infectious Diseases and Phychosomatic Medicine, Ghent, Belgium

## Abstract

**Background:**

The “Berlin patient” is the first patient functionally cured of HIV. He received stem cell transplantation from a homozygote CCR5-Δ32 donor. The reconstituted CD4^+^ T-cell population should be susceptible to infection with CXCR4-using viruses. According to gp120-V3 deep sequencing analysis of plasma-derived variants present before transplantation, the patient harbored a minority (2.9%) of viruses predicted to be CXCR4-tropic (geno2pheno_coreceptor_ FPR 10%). It remains puzzling why these variants failed to emerge post-transplant. We hypothesize that these CXCR4-predicted variants depend on CCR5 for replication.

**Methods:**

Patient-derived viral constructs were generated by cloning V3-sequences of the CXCR4-predicted viruses (pX1-pX7) and the dominant CCR5-predicted strain (pR5) into HXB2-ΔV3. As controls V3-sequences of HXB2 (cHXB2; CXCR4-tropic) and BaL (cBaL; CCR5-tropic) were cloned. Co-receptor preference was investigated in U-373-MAGI-cells expressing CD4^+^CCR5^+^ or CD4^+^CXCR4^+^, PBMCs from healthy donors and patient-derived post-transplant CCR5-Δ32 PBMCs.

**Results:**

Three pre-transplant CXCR4-predicted strains had an amino acid substitution in the V3 glycosylation-motif and one had a lysine at position 25, all associated with CXCR4-tropism. Five of the 7 viral clones were infectious. As expected cHXB2 infected CD4^+^CXCR4^+^-MAGI-cells and was inhibited by AMD-3100 (CXCR4-inhibitor) in donor PBMCs. Remarkably, the CXCR4-predicted viruses (FPR 2.7–9.3) depended on CCR5 for replication in MAGI-cells and were inhibited by maraviroc (CCR5-inhibitor) in donor PBMCs similar to pR5 and cBaL. As an ultimate proof it was shown that CXCR4-predicted strains could not replicate in the post-transplant derived CCR5-Δ32 PBMCs, whereas cHXB2 replication was observed.

**Conclusion:**

The minority population of CXCR4-predicted viral strains which the patient harbored pre-transplant were fully dependent on CCR5 for replication in vitro. This could explain lack of rebound after treatment discontinuation. This provides a strong rationale for the further development of CCR5-targeted gene therapy and suggests that successful reconstitution of CCR5-depleted immune system may work, even if there is some evidence of CXCR4-predicted variants.

# Sub article: THAA0102: Viral tissue reservoirs are determined early and little viral RNA is detected during suppression by three or four drug regimens in the macaque model

**Published:** 2012-10-22

**Authors:** C. Kline, J. Ndjomou, T. Franks, R. Kiser, V. Coalter, M. Piatak, J. Estes, J. Mellors, J. Lifson, Z. Ambrose

**Affiliations:** 1 University of Pittsburgh School of Medicine, Medicine, Pittsburgh, United States; 2 SAIC-Frederick, Inc., NCI Frederick, AIDS and Cancer Virus Program, Frederick, United States

## Abstract

**Background:**

Although HIV-infected individuals can suppress plasma viremia to undetectable levels with antiretroviral therapy, infected cells remain in the body and can contribute to viremia when therapy is discontinued. Macaque models allow investigators to more easily characterize viral reservoirs.

**Methods:**

Twelve male macaques were infected with RT-SHIV, an SIV virus containing HIV-1 reverse transcriptase, and monitored for plasma viremia and CD4 counts. After 10-14 weeks post-infection, 6 animals were not treated and 6 animals were treated for 17–20 weeks with 3 drugs (tenofovir, lamivudine, and efavirenz) or 4 drugs (tenofovir, lamivudine, efavirenz, and an integrase inhibitor). Viral RNA and viral DNA were measured longitudinally in the blood and at necropsy in over 20 different tissues by quantitative PCR and normalized for cellular RNA and DNA.

**Results:**

In untreated and treated animals, RT-SHIV DNA was highest in lymphoid and gastrointestinal tissues and very low to absent in the brain, genital tract, and kidney. The amount of viral DNA detected in multiple lymphoid tissues correlated with the level of plasma viremia 1 week post-infection. RT-SHIV RNA was abundant in the lymphoid tissues of untreated macaques with detectable viremia, but was detected variably in different regions of the gastrointestinal tract. Little or no viral RNA was detected in the tissues from animals after 17-20 weeks of therapy. There was no obvious difference in RT-SHIV RNA levels between animals treated with 3 or 4 drugs.

**Conclusion:**

Our results suggest that the majority of virally-infected cells are located in lymphoid tissues with variable levels in the gastrointestinal tract. The number of infected cells in these reservoirs correlates with viremia one week after infection, suggesting that viral reservoirs are seeded within days of infection. Little viral RNA is evident in tissue after suppressive therapy with either 3 or 4 antiretroviral drugs.

# Sub article: THAA0105: Characterization of persistent HIV-1 in a broad spectrum of CD4+ T cells isolated from peripheral blood and gut associated lymphoid tissue from patients on long-term suppressive therapy

**Published:** 2012-10-22

**Authors:** L. Josefsson, S. Eriksson, E. Sinclair, T. Ho, M. Killian, L. Epling, A. Tan, P. Lemey, N.R. Faria, W. Shao, P. Hunt, M. Somsouk, D. Douek, P. Bacchetti, L. Loeb, J. Custer, L. Poole, S. Deeks, F.M. Hecht, S. Palmer

**Affiliations:** 1 Karolinska Institutet, Department of Microbiology, Tumor and Cellbiology, Solna, Sweden; 2 The Swedish Institute for Communicable Disease Control, Department of Diagnostics and Vaccinology, Solna, Sweden; 3 University of California - San Francisco, Department of Medicine, San Francisco, United States; 4 KU Leuven, Rega Institute, Leuven, Belgium; 5 National Institiute of Cancer, Advanced Biomedical Computing Center, SAIC, Frederick, United States; 6 National Institute of Allergy and Infectious Diseases, National Institutes of Health, Immunology Laboratory, Vaccine Research Center, Bethesda, United States; 7 University of California - San Francisco, Department of Epidemiology and Biostatistics, San Francisco, United States

## Abstract

**Background:**

The role of ongoing virus replication in HIV persistence during long-term antiretroviral therapy is unknown. Since residual replication should result in detectable evolution, we investigated the degree of sequence evolution in blood-derived and rectal tissue-derived CD4+ T cells.

**Methods:**

Using single-genome and single-proviral sequencing techniques, we obtained 20-50 single viral genomes from pre-therapy plasma samples from 5 subjects who initiated therapy during acute infection and 3 subjects who initiated therapy during chronic infection. Pre-therapy plasma viral sequences were compared to single proviral HIV-1 genomes derived from HIV-1-infected T-cells (naïve, memory, central- and effector-memory) from peripheral blood (PB) and gut-associated lymphoid tissue (GALT) samples collected after 4-12 years of suppressive therapy. Maximum likelihood phylogenetic trees were constructed using the general time reversible model incorporating rate variation among sites. Evolutionary divergence was explored using root-to-tip analysis (Path-O-Gen).

**Results:**

The geometric mean infection frequency of memory and naïve CD4+ T-cells in the PB was 13- and 24-fold higher respectively in subjects treated during chronic compared to acute infection. This was also true for effector memory CD4+ T-cells from the GALT (6-fold higher). Phylogenetic analysis revealed clear evidence against any substantial evolution between the pre-therapy plasma-derived HIV RNA sequences and on-therapy intracellular HIV DNA sequences. Numerous intracellular HIV sequences identified after long-term therapy contained replication-incompetent virus. One patient had a predominant intracellular clone in both memory and effector memory T-cells containing a 380bp deletion after >9 years of therapy.

**Conclusion:**

Early initiation of effective therapy results in substantially lower reservoir size in blood and gut. The lack of HIV-1 genetic evolution in the HIV-1 infected CD4+ T-cell populations after years of therapy argues against virus replication as a major cause of persistence in these cell populations. The role of replication in other tissues and cell types however remains to be defined.

# Sub article: TUAA0103: Anti-APOBEC3G activity of HIV-1 Vif protein from elite controllers is attenuated compared to those from untreated chronic progressors or those from individuals with acute infection

**Published:** 2012-10-22

**Authors:** T. Kikuchi, Y. Iwabu, A. Kawana-Tachikawa, M. Koga, N. Hosoya, S. Nomura, Z.L. Brumme, H. Jessen, A. Kelleher, M. Markowitz, F. Pereyra, A. Trocha, B.D. Walker, A. Iwamoto, K. Tokunaga, T. Miura

**Affiliations:** 1 The Institute of Medical Science, the University of Tokyo, Tokyo, Japan; 2 National Institute of Infectious Diseases, Tokyo, Japan; 3 Simon Fraser University, Burnaby, Canada; 4 Jessen Praxis, Berilin, Germany; 5 University of New South Wales, Sydney, Australia; 6 Aaron Diamond AIDS Research Center, New York, United States; 7 MIT and Harvard, Ragon Institute of MGH, Charlestown, United States; 8 Nagasaki University, Nagasaki, Japan

## Abstract

**Background:**

HIV-1-infected individuals who control viremia to below the limit of detection without antiviral therapy have been termed elite controllers (EC). Functional attenuation of some HIV-1 proteins has been reported in EC. However, little is known about role of the HIV-1 accessory protein Vif function in EC, which enhances HIV-1 infectivity through APOBEC3G degradation. In this study, the anti-APOBEC3G function of Vif was compared between EC, chronic progressors (CP) and individuals with acute infection (AI).

**Methods:**

Forty-nine EC, 49 CP and 44 AI were studied. *vif* genes were amplified by nested RT-PCR using concentrated plasma. To compare anti-APOBEC3G activity of Vif proteins among those groups, VSV-G-pseudotyped viruses were generated by co-transfecting 293T cells with expression plasmids encoding patient-derived Vif, APOBEC3G, VSV-G, together with a *vif/env*-deficient HIV-1 proviral DNA clone carrying a luciferase reporter gene. VSV-G-pseudotyped viruses were normalized for p24 antigen and used to infect 293T cells and luciferase activity was measured at 48 h postinfection.

**Results:**

Anti-APOBEC3G activity of Vif from EC was significantly reduced compared to those from CP or AI (Figure 1). These results remained significant after excluding individuals expressing protective HLA alleles B*27 and/or B*57. No significant difference was observed between CP and AI. Significant differences in amino acid usage in *vif* genes were found at 7 residues between CP and EC, and at 13 residues between AI and EC. However, there were no common polymorphisms (away from consensus B) that could explain reduced anti-APOBEC3G activity of Vif derived from EC.

**Conclusion:**

Anti-APOBEC3G activity of Vif proteins derived from EC was reduced. This reduced activity was independent of presence or absence of known protective HLA alleles. Common Vif mutations in EC unlikely explain the observed reduction; rather it might be attributable to unique mutations to each EC Vif protein.

**Figure 1:** Comparison of anti-APOBEC3G activity of Vif From AI, CP and EC patients. Each dot represents mean RLU of each patient.

# Sub article: TUAA0203: CBF-1 induces both establishment and maintenance of HIV latency via recruiting PcG corepressor complex at LTR

**Published:** 2012-10-22

**Authors:** M. Tyagi

**Affiliations:** 1 George Mason University, Manassas, United States

## Abstract

**Background:**

Repressive epigenetic modifications have been shown to induce and maintain HIV latency; however underlying molecular mechanisms are not yet clear. We have previously demonstrated the critical role of CBF-1 (Latency-C-promoter binding factor 1) in establishing repressive chromatin structures at HIV LTR during latency establishment. The knockdown of CBF-1 results in the reactivation of latent proviruses and overexpression of CBF-1 facilitates latency establishment. Here we extend these studies to show that multiple repressive epigenetic modifications that CBF-1 induces are the result of recruitment of Polycomb Group (PcG) corepressor complex at HIV LTR by CBF-1.

**Methods:**

Both transformed and primary T cells were infected with lentiviral vectors expressing Tat *in cis* to study the underlying molecular mechanisms regulating HIV latency and via running various molecular assays, including Chromatin Immunoprecipitation (ChIP) assays.

**Results:**

In this study, we demonstrate that CBF-1 induces repressive chromatin structures at HIV LTR by recruiting Polycomb Group (PcG) corepressor complex at HIV LTR. The knockdown of endogenous CBF-1 results in the dissociation of PcG complex components from HIV LTR. Furthermore, knockdown of the individual components of PcG complex leads to the reactivation of latent HIV proviruses demonstrating the direct role of PcG complex in establishing HIV latency. Overall our results demonstrate that the CBF-1 induced various epigenetic modifications are the result of recruitment of Polycomb Group (PcG) corepressor complex at HIV LTR, which carry a variety of epigenetic factors that repress HIV gene expression via generating several layers of repressive epigenetic modifications.

**Conclusion:**

We have established that analogous to the transformed T cell lines in primary T cells CBF-1 induced repressive chromatin structures play important role in establishing HIV latency. Additionally by recruiting PcG corepressor complex at LTR, CBF-1 not only facilitates HIV latency establishment also play critical role in maintaining and stabilizing the latent proviruses.

# Sub article: THAA0103: Distribution of the HIV reservoir in patients spontaneously controlling HIV infection after treatment interruption

**Published:** 2012-10-22

**Authors:** C. Bacchus, L. Hocqueloux, V. Avettand-Fenoël, A. Saez-Cirion, A. Mélard, B. Descours, A. Samri, C. Blanc, B. Autran, C. Rouzioux, VISCONTI and ALT ANRS study groups

**Affiliations:** 1 Cellular and Tissular Immunology Laboratory, Pierre and Marie Curie UniversityPitié-Salpêtrière Hospital, INSERM UMR-S 945, Paris, France; 2 Infectious and Tropical Diseases Department, Regional Hospital, Orléans, France; 3 Virology Laboratory, René Descartes University, Necker Hospital, Paris, France; 4 Institut Pasteur, Unité de Régulation des Infections Rétrovirales, Paris, France; 5 Human Genetic Institute, Molecular Virology Laboratory, CNRS UPR1142, Montpellier, France; 6 Flow Cytometry Platform CyPS, Pierre and Marie Curie University, Pitié-Salpêtrière Hospital, Paris, France

## Abstract

**Background:**

Virological and Immunological Studies in CONtrollers after Treatment Interruption (VISCONTI) are required to understand the benefits of an early treatment at acute HIV-1 infection on the HIV reservoir. We studied the distribution, magnitude and inducibility of the HIV reservoir in VISCONTI patients.

**Methods:**

The prospective VISCONTI study included twelve patients controlling HIV for a median of 76[IQR:67.5–84.5] months after interruption of a 3[IQR:1.7–5.9] years long HAART initiated within 10 weeks post-infection. Circulating resting CD25-CD69-HLADR- CD4+T cell subsets were sorted as naive (TN), central-memory (TCM), transitional-memory (TTM) and effector-memory cells (TEM) for further cell-associated HIV-DNA quantification by ultrasensitive real-time-PCR, and viral inducibility by culture with anti-CD3/anti-CD28/IL-2/IL-7. Reservoir distribution was compared to the one observed in 8 untreated Elite-Controllers for whom 90% of HIV-RNA measures was undetectable (below 200 copies) over 12[9–14] years.

**Results:**

In the VISCONTI group, activated CD4+T cells had significantly higher HIV-DNA levels than resting ones (median 2.7[IQR:2.4–3.4] and 2[IQR:1.8–2.5] log copies/million cells, p=0.005). HIV-DNA was detected in all subsets from all patients except for 8 out of 12 TN-sorted cells, which were 10 fold less infected than all memory subsets (median TN:1.5[IQR:1.2–1.6], TCM:2.5[IQR:1.8–2.9], TTM:2.6[IQR:2.2–2.8] and TEM:2.4[IQR:2–2.8] log copies/million cells, p< 0.007). TTM was the major subset contributing to 56% of this reservoir. The same HIV reservoir characteristics were observed in Elite-Controllers in term of magnitude and distribution, except that both TCM and TTM equally contributed to the Elite-Controllers HIV reservoir. The VISCONTI HIV reservoir was inducible after TCR-stimulation in all sorted memory subsets from all patients, except in TN where no virus was recovered in 6 out of 8 patients.

**Conclusion:**

In VISCONTI patients, treatment initiated at primary HIV-1 infection leads, after treatment interruption, to a low -but inducible- durable HIV reservoir distributed mainly in short-lived memory CD4+T cells that mimicks the natural distribution observed in Elite-Controllers.

# Sub article: MOPDA0105: Immune and inflammatory gene expression in the periphery and CNS of cART-treated SIV-infected macaques

**Published:** 2012-10-22

**Authors:** M.C. Zink, D.R. Graham, L. Gama, S.E. Queen, K.A. Meulendyke, J.L. Mankowski, J.E. Clements

**Affiliations:** 1 Johns Hopkins School of Medicine, Baltimore, United States

## Abstract

**Background:**

A major hurdle toward curing HIV is the establishment of long-lived latent reservoirs such as the CNS. Using an SIV model of HIV infection we examined the effect of combination antiretroviral therapy (cART) on immune and inflammatory gene expression in the periphery and CNS that leads to virus downregulation.

**Methods:**

To examine the effect of cART on acute and long-term systemic and CNS immunopathogenesis, groups of SIV-infected macaques were untreated and euthanized at 21 postinoculation (dpi) or end stage disease, or treated with cART starting at 4 or 12 dpi and euthanized at 21 or 175 dpi, respectively. RNAs for immune and inflammatory genes were quantified in the spleen and brain by non-amplification Nanostring technology or by qRT-PCR. SIV replication and viral DNA were also measured.

**Results:**

cART initiation at 4 or 12 dpi did not prevent SIV seeding of the brain; brain viral DNA levels were the same as in untreated animals. cART treatment initiated at 4 dpi had little effect on peripheral and CNS immune and inflammatory gene expression profiles as compared to responses mounted in untreated macaques after acute infection, as indicated by similar levels of IL17A, IL17F, and CCL5 in the periphery and IL-6, IFNß, and TNFa in the CNS.

**Conclusion:**

The CNS is a latent reservoir for SIV that is seeded early after infection regardless of cART initiation at 4 or 12 dpi. The innate and adaptive immune responses are nearly as effective as early cART treatment at returning the host to peripheral and CNS immune homeostasis by 21 dpi. However, at the same time those responses likely promote the establishment of latent reservoirs by suppressing viral replication, not eliminating the reservoir.

# Sub article: MOPDA0101: Toll-like receptor (TLR) 9 variant is associated with mother-to-child transmission (MTCT) of HIV-1 and TLR9 and TLR8 variants are associated with peak viral load in HIV-1-positive infants

**Published:** 2012-10-22

**Authors:** K. Beima-Sofie, A. Bigham, J.R. Lingappa, D. Wamalwa, R.D. Mackelprang, E. Maleche-Obimbo, B. Richardson, G. John-Stewart

**Affiliations:** 1 University of Washington, Public Health Genetics, Seattle, United States; 2 University of Michigan, Anthropology, Ann Arbor, United States; 3 University of Washington, Medicine, Seattle, United States; 4 University of Washington, Global Health, Seattle, United States; 5 University of Washington, Pediatrics, Seattle, United States; 6 University of Nairobi, Nairobi, Kenya; 7 University of Washington, Seattle, United States; 8 University of Washington, Biostatistics, Seattle, United States; 9 University of Washington, Epidemiology, Seattle, United States

## Abstract

**Background:**

Toll-like receptors (TLRs) are critical proteins of the innate immune system. We evaluated association of single nucleotide polymorphisms (SNPs) in 6 *TLR* and 2 *TLR*-associated genes with infant HIV-1 acquisition and progression.

**Methods:**

HIV-outcomes were assessed from birth to 1-year of age among infants from a Kenyan perinatal cohort in which HIV-infected women were enrolled during pregnancy and received short-course zidovudine. Infants were genotyped for 6 candidate and 118 haplotype-tagging polymorphisms in *TLRs* 2, 3, 4, 7, 8, and 9, *MyD88* and *TIRAP*, and 144 ancestral informative markers. Cox proportional hazards and linear regression were performed to assess *TLR* polymorphism associations with HIV-1 acquisition, peak HIV-1 RNA levels, and infant mortality. Sex-stratified analyses of TLR7 and TLR8 were conducted due to their X-chromosome location.

**Results:**

Among 368 mother-infant pairs, 56 (15%) infants acquired HIV-1 by month 1 and 17 (4.6%) between 1 and 12 months. Infants with the *TLR9* 1635A (rs352140) variant were more likely to acquire HIV by 1 month (HR=1.49, 95% confidence interval [CI] =1.04–2.38, p=0.028) and by month 12 (HR=1.40, CI=1.02–1.92, p=0.038) in additive models adjusted for maternal plasma HIV-1 viral load (VL) and genetic ancestry. Among 56 HIV-1 infected infants infected at<1 month, peak VL was 6.8 log_10_ c/ml. The *TLR9* 1635A allele was associated with a 0.40 log_10_ c/ml decrease in peak VL (p=0.002); female infants with the *TLR8* 1G (rs3764880) variant had a 0.62 log_10_ c/ml increase in peak VL (p=0.001). No variants were significantly associated with mortality in infected infants.

**Conclusion:**

This study is the first to evaluate the association between *TLR* polymorphisms and HIV-related outcomes in a perinatal African cohort and confirms *TLR9* associations previously observed in Caucasians. Defining the role of *TLR* polymorphisms in HIV-1 transmission and progression may inform future prevention strategies that exploit the innate immune response.

# Sub article: MOPDA0102: Polymorphisms in TLR2 and TLR7 are associated with plasma HIV-1 RNA set-point in an African heterosexual cohort

**Published:** 2012-10-22

**Authors:** R. Mackelprang, A. Bigham, C. Celum, G. de Bruyn, K. Beima, G. John-Stewart, A. Ronald, N. Mugo, A. Rainer, K. Buckingham, M. Bamshad, J. Mullins, J. McElrath, J. Lingappa

**Affiliations:** 1 University of Washington, Seattle, United States; 2 University of Michigan, Ann Arbor, United States; 3 University of the Witwatersrand, Johannesburg, South Africa; 4 University of Manitoba, Winnipeg, Canada; 5 University of Nairobi, Kenyatta National Hospital, Nairobi, Kenya

## Abstract

**Background:**

The Toll-like receptor (TLR) genes mediate the innate response to viral infections and may impact HIV-1 pathogenesis. We evaluated TLR polymorphisms for association with plasma HIV-1 RNA set-point in HIV-1 infected individuals from East and Southern Africa.

**Methods:**

Analyses included prospective data and DNA from 500 Africans with heterosexually-acquired HIV-1 (125 incident, 375 prevalent). For incident HIV-1, set-point was defined as the median plasma HIV-1 RNA level ≥4 months after the estimated infection date. For prevalent HIV-1, set-point was the average of ≥2 consecutive plasma HIV-1 RNA measurements before ART initiation or CD4 decline to<200 cells/mm^3^. Genotyping was performed for 124 single nucleotide polymorphisms (SNPs) from 6 TLR and 2 TLR-associated signaling genes (TIRAP and MYD88) and 144 ancestral informative markers. These included 8 candidate SNPs previously associated with HIV-1, and 115 haplotype tagging SNPs (tagSNPs) representing common variation across TLR genes. Associations were determined using linear regression with adjustment for sex, age, and population stratification, and Bonferroni correction.

**Results:**

Among 492 HIV-1 infected individuals who passed quality control, the median HIV-1 set-point was 4.6 (IQR: 3.8–5.0) log_10_ copies/mL and did not differ between seroprevalent and seroincident participants. *TLR2-*r*s3804100* (minor allele frequency [MAF]=0.053), *a* candidate C-T synonymous SNP located in exon 1 was associated with an average 0.37 (95% confidence interval [CI]: 0.10, 0.65, p=0.007) log_10_ copies/ml increased set-point. *TLR7-rs179012*, a haplotype-tagging SNP located in intron 1 was associated with a 0.31 (95% CI: 0.47, 0.14, Bonferroni-adjusted p=0.032) log_10_ copies/ml decrease in HIV-1 set-point.

**Conclusion:**

These are the first associations between TLR polymorphisms and plasma HIV-1 RNA level reported among African populations. *TLR2 rs3804100* has been previously linked with more rapid disease progression in Caucasians. Our finding of *TLR7 rs179012*and improved control of infection has not been previously reported. Further study of these SNPs may improve understanding of HIV-1 pathogenesis.

# Sub article: MOPDA0103: Association of polymorphisms in the regulatory region of cyclophilin A gene (PPIA) with disease progression and gene expression levels

**Published:** 2012-10-22

**Authors:** P. Madlala, R. Singh, L. Werner, S. Sibeko, P. An, S.S.A. Karim, C.A. Winkler, T. Ndung'u

**Affiliations:** 1 University of KwaZulu-Natal, Nelson R. Mandela School of Medicine, HIV Pathogenesis Programme (HPP), Durban, South Africa; 2 University of KwaZulu-Natal, Genetics, Pietermaritzburg, South Africa; 3 University of KwaZulu-Natal, Nelson R. Mandela School of Medicine, Centre for the AIDS Programme of Research in South Africa (CAPRISA), Durban, South Africa; 4 National Cancer Institute-Frederick, Basic Research Laboratory, Frederick, United States

## Abstract

**Background:**

Human cyclophilin A (CypA) encoded by peptidyl prolyl isomerase A gene (PPIA), is an important cellular co-factor for efficient human immunodeficiency virus type 1 (HIV-1) infection. In this study we investigated the effect of genetic variation in the regulatory region of PPIA on HIV-1 disease progression and CypA mRNA expression levels in HIV-1 South African cohorts.

**Methods:**

A total of 603 black South African participants from these cohorts were genotyped for single nucleotide polymorphism (SNP) A1650G in the regulatory region of CypA using PCR-RFLP. 247 (195 HIV-1 seronegative participants [SNs] and 52 primary infected participants [SPs]) participants were from the CAPRISA acute infection (AI) 002 cohort and 356 HIV-1 chronically infected participants were from the Sinikithemba cohort. CypA mRNA expression was quantified in 30 SNs and 28 SPs from the CAPRISA AI 002 cohort by real-time RT-PCR. Lastly, we assessed the effect of SNPA1650G on viral (NL4.3) replication in PBMCs isolated from HIV-1 negative individuals.

**Results:**

The minor allele (G) of SNP A1650G (referred to as 1650G) was significantly associated with higher viral load (p<0.01) and lower CD4^+^ T cell count (p< 0.01) during primary HIV-1 infection. Interestingly, the1650G was associated with rapid CD4^+^ T cell decline during chronic infection (p=0.01). The 1650G was also significantly associated with higher CypA mRNA expression levels (p<0.01). PBMCs isolated from participants harboring the 1650G supported higher levels of NL4.3 replication ex vivo.

**Conclusion:**

Our results suggest that higher expression levels of CypA mRNA enhance HIV-1 replication in a South African population. The results demonstrate the clinical relevance of CypA and provide additional in vivo validation of the CypA as a pertinent target for therapeutic intervention. This study supports the development of small molecule inhibitors against CypA and HIV-1 interaction.

# Sub article: WEPDA0101: HLA class I associations with rates of HIV-1 seroconversion and disease progression in the Pumwani sex worker cohort

**Published:** 2012-10-22

**Authors:** T. Peterson, J. Kimani, C. Wachihi, T. Bielawny, L. Mendoza, S. Thavaneswaran, M. Narayansingh, T. Kariri, B. Liang, E. Ngugi, F. Plummer, M. Luo

**Affiliations:** 1 National Microbiology Laboratory, HIV and Human Genetics, Winnipeg, Canada; 2 University of Nairobi, Department of Medical Microbiology, Nairobi, Kenya; 3 University of Manitoba, Department of Medical Microbiology, Winnipeg, Canada; 4 National Microbiology Laboratory, Winnipeg, Canada; 5 Universiy of Nairobi, Department of Community Health, Nairobi, Kenya

## Abstract

**Background:**

Class I Human Leukocyte Antigens (HLA) play an important role in the adaptive immune response by presenting antigens to CD8+ T-cells. Previous studies have reported multiple HLA associations with rates of disease progression in HIV infected individuals, while few class I associations with resistance or susceptibility to HIV-1 infection have been reported.

**Methods:**

HLA-A, -B, and -C were typed for more than 1000 women enrolled in the Pumwani sex worker cohort using a sequence-based typing method. Kaplan-Meier analysis was used to identify alleles influencing seroconversion and disease progression to AIDS (CD4+ decline to<200/mm3).

**Results:**

A*01 (*P*=0.020), C*06:02 (*P*=0.042) and C*07:01 (*P*=0.050) are independently associated with protection from seroconversion. Women with any of these alleles are better protected from seroconversion (*P*=0.003, OR:1.988, 95% CI:1.267–3.122) than those without them. Conversely, A*23:01 (*P*=0.004), B*07:02 (*P*=0.003) and B*42:01 (*P*=0.025) are independently associated with rapid seroconversion. Women with any of these alleles are twice as likely to seroconvert (*P*=0.002, OR:0.486, 95% CI:0.304–0.775). The effect of beneficial alleles in protection from seroconversion is more than three fold when compared with those with susceptible alleles (*P*=0.00004, OR:3.636, 95% CI:1.930–6.852). B*14(*P*=0.003) and B*57:03(*P*=0.012) are independently associated with slower progression to AIDS, while B*53:01(*P*=0.035) is associated with rapid CD4+ T-cell decline. Women with B7 supertype rapidly progressed to AIDS and individuals homozygous for this supertype fared even worse (*P*=0.004).

**Conclusion:**

Understanding why these HLA class I alleles are associated with protection/susceptibility to HIV-1 acquisition and disease progression could contribute to the development of effective prophylactic and therapeutic vaccines for HIV-1.

# Sub article: WEPDA0206: TRIM22 repression of HIV-1 transcription is mediated by interaction with SP1

**Published:** 2012-10-22

**Authors:** A. Kajaste-Rudnitski, S. Marelli, G. Poli, B. Berkhout, A.T. Das, E. Vicenzi

**Affiliations:** 1 San Raffaele Scientific Institute, Milan, Italy; 2 Vita-Salute San Raffaele University, Milan, Italy; 3 University of Amsterdam, Department of Medical Microbiology, Amsterdam, Netherlands

## Abstract

**Background:**

We have recently shown that the IFN-inducible Tripartite motif-containing protein 22 (TRIM22) suppresses basal HIV-1 LTR-mediated transcription through a Tat-and NF-κB-independent mechanism [Kajaste-Rudnitski et al., *J Virol* 2011, 85(10):5183–96]. In the absence of Tat/TAR RNA complex, HIV-1 transcription can occur through the positive elongation factor (P-TEF) b function and Sp1. We have here investigated whether TRIM22 interferes with Sp1-mediated transcriptional activation of the HIV-1 LTR.

**Methods:**

293T cells, devoid of endogenous TRIM22, were transfected with increasing amounts of a TRIM22-expressing plasmid together with a fixed amount of an HIV-1 LTR reporter construct that contains a TAR sequence unresponsive to Tat and two tet-O motifs that bind to rtTA in the presence of doxycycline (Dox). Constructs containing the deletion of one, two or three Sp1 sites were also tested.

**Results:**

TRIM22 efficiently inhibited Dox-induced WT HIV-1 LTR transcription. Interestingly, this inhibitory effect was progressively lost with the LTR reporters lacking one, two or all three Sp1 binding sites, respectively. In line with these observations, addition of three Sp1 sites into a reporter construct based on a CMV-derived promoter element, normally insensitive to TRIM22, renders it susceptible to TRIM22 transcriptional repression, indicating that TRIM22 could have a broader regulatory role in Sp1-mediated gene expression. Although TRIM22 does not alter overall Sp1 expression levels when transfected into 293T cells, immunoprecipitation experiments performed on 293T cells transfected with a FLAG-tagged TRIM22 revealed that TRIM22 directly interacts with Sp1.

**Conclusion:**

These results suggest that TRIM22 may inhibit HIV-1 LTR-driven transcriptional initiation through interference with the Sp1-mediated signaling and activation of early HIV-1 gene expression, rendering it an attractive candidate for novel therapeutic approaches.

# Sub article: THPDA0204: Apobec3G levels are inversely associated with resting CD4+ T memory cell integrated provirus *in vivo* and infectivity of reactivated HIV-1 *ex vivo*

**Published:** 2012-10-22

**Authors:** M. De Pasquale, Y. Kourteva, T. Allos, R. D'Aquila

**Affiliations:** 1 Vanderbilt University School of Medicine, Infectious Diseases, Nashville, United States; 2 Vanderbilt University School of Medicine, Nashville, United States

## Abstract

**Background:**

We hypothesized that APOBEC3G (A3G) was associated with provirus burden in resting memory CD4+ T cells, and infectivity of HIV produced from them.

**Methods:**

Cells from antiretroviral-naïve, long-term non-progressor (LTNP, n=7) and HAART-suppressed (HS, n=11) subjects were negatively selected from PBMCs (Robo-Sep). Sorting (FACS Aria, BD) separated activated cells (CD25+, CD69+, CD38+ and HLA-DR+) from resting central memory (Tcm: CCR7+, CD45RO+), resting effector memory (Tem: CCR7-, CD45RO+, which includes resting transitional memory) and resting naïve (CCR7+, CD45RO) T cells. A3G was quantified by immunoblotting (Odyssey, Li-Cor). Provirus was quantified by *alu*-PCR. Reactivated HIV was recovered from cells treated *ex vivo* with anti-CD3,8 bispecific monoclonal antibody and IL2. Virus p24 levels in culture supernatants were quantified by ELISA. Infectivity of virus produced from *ex vivo* activated cells was assessed using TZM-bl indicator cells.

**Results:**

Tcm and Tem from LTNP had less provirus *in vivo* than the same cell type from HS subjects (p= 0.01 for Tcm and 0.02 for Tem). A3G protein levels were higher in Tcm and Tem from the LTNP, in comparison to Tcm and Tem from the HS, subjects (p=0.02 for Tcm and p=0.02 for Tem). Virions were recovered from *ex vivo* activated Tcm from 5 of the HS subjects. Infectivities of normalized virion amounts were inversely associated with the A3G levels in virions produced from them (Spearman r=-0.99, p=0.01)

**Conclusion:**

Resting central and effector memory CD4+ T cells from LTNP had less provirus and higher levels of A3G protein than the same cell types from HS subjects. Infectivity of HIV reactivated *ex vivo* from Tcm of HS subjects was inversely associated with virion A3G levels. A3G appears able to restrict viral spread from proviruses reactivated from resting memory CD4+ T cells, suggesting that improving this restriction may contribute to 'curing' HIV.

# Sub article: WEPDA0201: Integration of global techniques to implicate post-transcriptional regulation in HIV infection

**Published:** 2012-10-22

**Authors:** J. Blackinton, M. Thompson, N. Mukherjee, S. Freel, G. Tomaras, J. Keene

**Affiliations:** 1 Duke University, Durham, United States

## Abstract

**Background:**

The immediate and early dynamic cellular response to an HIV virion entering the cell is not well understood, particularly at the post-transcriptional level where known rapid immune responses occur. Several RNA-binding proteins (RBPs) are known to be important in infection, including HuR, which is known to bind to and regulate the mRNA transcripts of several of the known early entry co-factors such as PPIA, TRIM5, APOBEC3G and CUL5.

**Methods:**

We have previously integrated three global methods to measure post-transcriptional regulation in Jurkat T cell activation and examine relationships between ribonucleoprotein (RNP) interactions, transcription, RNA stability and translation. Dynamic changes in association with HuR caused measurable effects on RNA stability and translation of mRNAs. Transcripts that increased in association with HuR during activation showed increased stability and translation while those that decreased show decreased stability and translation. Furthermore, these changed transcripts fell into relevant functional groups, where cell cycle regulators were largely decreased in association, stability and translation, and DNA/RNA regulators were largely increased in association, stability and translation.

**Results:**

To test the effect of post-transcriptional regulation in HIV infection, we used the C8166 T cell line for a near complete and synchronous infection and measured global changes in RNA stability and rates of transcription using 4-thiouridine stability profiling at five time points over the initial 24 hours of infection. Simultaneously, we measured dynamic changes in mRNA association with HuR during the same infection time points. Comparing the two datasets, we observed several mRNA transcripts, including myc mRNA, a known target of HuR, showing dynamic regulation at the level of stability during the infection time course.

**Conclusion:**

Elucidation of these events supports the involvement of the post-transcriptional response early in HIV infection and suggests that potentially modifying or amplifying the post-transcriptional response may be able to reduce the efficiency of infection.

# Sub article: MOAA0101: The effect of liquefaction on the ability of semen and semen amyloid fibrils to enhance HIV infection

**Published:** 2012-10-22

**Authors:** N. Roan, J. Muller, A. Gawanbacht, O. Zirafi, S. Chu, F. Kirchhoff, J. Munch, W. Greene

**Affiliations:** 1 The J. David Gladstone Institutes, Gladstone Institute of Virology and Immunology, San Francisco, United States; 2 University of California at San Francisco, Department of Urology, San Francisco, United States; 3 Institute of Molecular Virology, Ulm University Medical Center, Ulm, Germany; 4 University of California at San Francisco, Departments of Medicine, Microbiology and Immunology, San Francisco, United States

## Abstract

**Background:**

Semen, the most common vector for HIV transmission, enhances HIV infection *in vitro*. We recently identified amyloid fibrils comprised of fragments from semenogelins, the predominant component of the semen coagulum. Semenogelin amyloids interact with virions (Figure 1) and potently enhance HIV infection of target cells. During semen liquefaction, semenogelins are fragmented and eventually completely degraded by PSA. We hypothesized that if semenogelins are important for the viral enhancing activity of semen, then the change in the levels of these proteins during liquefaction should affect the ability of semen to enhance infection.

**Methods:**

Seminal fluid liquefied for different amounts of time were assayed for viral enhancing activity and semenogelin levels in the absence or presence of PSA inhibitors. We also tested the effect of purified PSA on the activity of amyloids formed from chemically-synthesized semenogelin peptides.

**Results:**

The viral enhancing activity of semen decreases with increasing liquefaction time in a manner that parallels the degradation of semenogelins. Semenogelin degradation and loss of viral enhancing activity in semen are both prevented in the presence of a PSA inhibitor. Finally, PSA specifically cleaves and inhibits the viral enhancing activity of semenogelin fibrils.

**Conclusion:**

Seminal fluid's viral enhancing activity is retained for several hours and then progressively decreases during prolonged liquefaction. We found a correlation between activity and semenogelin levels during liquefaction. These findings underscore the importance of semenogelins for the viral enhancing activity of semen. We also provide evidence that PSA directly regulates the activity of semenogelin fibrils, suggesting that these amyloids are regulated by liquefaction. As such, mechanisms to enhance the natural liquefaction process may be a useful approach to limit the ability of semen to enhance viral transmission.

**Figure 1:** Roan et al. Figure.

# Sub article: MOAA0103: Impact of depot medroxyprogesterone (DMPA) on human vaginal leukocytes and HIV-1 target cells

**Published:** 2012-10-22

**Authors:** N. Chandra, A. Thurman, S. Anderson, T. Cunningham, C. Mauck, G. Doncel

**Affiliations:** 1 CONRAD, Eastern Virginia Medical School, Norfolk, United States; 2 Eastern Virginia Medical School, School of Public Health, Norfolk, United States

## Abstract

**Background:**

The relationship between exogenous contraceptive hormones and permissiveness of the female genital tract to human immunodeficiency virus type 1 (HIV-1) remains the subject of intense debate, due to a recent study showing a two-fold increase in the rate of acquisition and transmission in serodiscordant couples using depot medroxyprogesterone acetate (DMPA)[1]. In order to better characterize the effect of DMPA on the vagina, we compared the leukocyte populations and density of epithelial junction proteins in vaginal tissue biopsies of women at baseline (during both the follicular and luteal phases of the menstrual cycle) and 12 weeks after receiving one DMPA injection.

**Methods:**

Vaginal biopsies were obtained from 20 healthy women in the follicular and luteal phases of the menstrual cycle, and approximately 12 weeks after receiving a 150 milligram intramuscular injection of DMPA. Leukocyte populations, activation phenotype and epithelial thickness and tight junction and adherens proteins were measured by immunohistochemistry and integrated optical density. Statistical analyses were performed using Wilcoxon signed rank tests.

**Results:**

After DMPA administration, CD3, CD8, CD45, CD68, HLA-DR and CCR5 bearing lymphocytes were all significantly (p<0.05) increased in vaginal tissues, compared to the follicular and or luteal phases. There were no significant differences in vaginal leukocyte populations between the follicular and luteal phases of the control cycle (p>0.05). Epithelial thickness and tight junction and adherens proteins were not statistically different between sampling times (p>0.05).

**Conclusion:**

After exposure to DMPA, vaginal leukocyte populations significantly increase in the vaginal mucosa. In absence of changes in epithelial integrity, the increase in vaginal T cells, activation markers, and HIV-1 receptors point to a possible immunological basis for the observed effects of DMPA on HIV-1 acquisition and transmission in women.

# Sub article: MOAA0105: HIV-1 transmission from semen to cervicovaginal tissue *ex vivo*

**Published:** 2012-10-22

**Authors:** A. Introini, C. Vanpouille, A. Lisco, J.-C. Grivel, A. Munawwar, S. Singh, L. Margolis

**Affiliations:** 1 National Institute of Child Health and Human Development, National Institutes of Health, Bethesda, United States; 2 All India Institute of Medical Sciences, New Delhi, India

## Abstract

**Background:**

Semen is the main carrier of sexually transmitted viruses, including HIV-1. However, semen of HIV-infected men is not merely a passive transporter of HIV-1 but, because of its richness in biologically-active compounds, including chemokines, may facilitate HIV-1 transmission. To test this hypothesis and to study HIV-1 transmission under controlled conditions, we evaluated HIV-1 loads and the cytokine milieu in semen and blood from infected men as well as, the role of these cytokines in HIV transmission to cervicovaginal tissue *ex vivo*.

**Methods:**

We measured 21 cytokines/chemokines with a multiplex bead assay and evaluated the loads of HIV-1 in seminal and blood plasmas from 50 HIV-1-infected and 28 uninfected Indian men.

**Results:**

We found that semen and blood are two separate immunological compartments, in which concentrations of cytokines and loads of coinfecting herpesviruses are profoundly different. Upon HIV infection, the levels of blood and semen cytokines were significantly altered, thus facilitating cytokine network compartmentalization. HIV-1 infection changes the seminal cytokine spectrum by upregulating 16 of the 21 measured cytokines, while in blood 2 cytokines were downregulated and 7 were upregulated. One of the most prominent cytokine in semen was IL-7, which was significantly upregulated in semen of HIV-1-infected individuals. IL-7 in concentrations similar to that in semen of HIV-1-infected individuals facilitates HIV-1 infection in cervicovaginal tissue *ex vivo*. This facilitation is associated with a suppression of apoptosis of infected CD4 T cells.

**Conclusion:**

HIV-1 infection results in an aberrant production of cytokines, changing the seminal cytokine network. The altered seminal milieu is an important determinant of HIV-1 sexual transmission: Cytokines altered by seminal infection facilitate HIV-1 transmission to cervicovaginal tissue *ex vivo*. Cervicovaginal tissue infected *ex vivo* provides a platform to study the mechanisms of this phenomenon and to develop new preventive strategies by targeting the seminal microenvironment.

# Sub article: MOLBA03: HIV-1 female-to-male sexual transmission: evaluation of circumcised and uncircumcised penile tissue

**Published:** 2012-10-22

**Authors:** M. Dinh, M. Anderson, C. Gioia, M. McRaven, Z. Okocha, G. Cianci, T. Hirbod, G. Kigozi, J. Prodger, R. Kaul, X. Kong, R. Gray, T. Hope

**Affiliations:** 1 Northwestern University Feinberg School of Medicine, Cell and Molecular Biology, Chicago, United States; 2 Karolinska Institutet, Stockholm, Sweden; 3 Rakai Health Sciences Program, Entebbe, Uganda; 4 University of Toronto, Toronto, Canada; 5 Johns Hopkins Bloomberg School of Public Health, Baltimore, United States

## Abstract

**Background:**

Male circumcision has been shown to decrease rates of HIV acquisition in African men. The STEP vaccine trial also demonstrated that vaccinated, uncircumcised men were at increased risk for HIV acquisition. We sought to identify the mode(s) by which HIV infection may occur in uncircumcised men.

**Methods:**

Foreskins were obtained from consenting male donors receiving prophylactic male circumcision in Rakai, Uganda. Whole penile specimens were obtained from tissue donation organizations (ScienceCare and NDRI). Using fluorescent immunohistochemistry, foreskin keratin layers were labeled with filaggrin and involucrin markers. Penile tissues were incubated with *ex vivo* with photo-activatable GFP-linked-Vpr HIV_Bal_ for 4 hours, snap-frozen, and cryosections stained for target cells and keratin. Images were obtained with epifluorescent microscopy and analyzed for keratin thickness, viral particles, and viral penetration into penile epithelia.

**Results:**

We found no significant differences between inner and outer foreskin keratin layers from 19 foreskin samples obtained in Uganda, indicating that reduced keratin thickness is not likely to make the inner foreskin more susceptible to HIV. Preliminary data from whole penile specimens (uncircumcised *n*=7, circumcised *n*=7) shows no significant difference in number of visualized virions per image captured, but more virions entering uncircumcised as compared to circumcised glans tissue (uncircumcised:circumcised=2:1). Virions were found at distances from the epithelial surface (mean±SD=33.5±22.3 µm) in the range where CD4+ cells are also localized (mean±SD=53.6 ±32.3 µm), in the absence of trauma to the epithelium. Finally, we visualize virions interacting with the male urethral pseudo-stratified columnar epithelia, though to a lesser degree than seen with stratified squamous epithelia (*n*=5, glans:urethra=2:1).

**Conclusion:**

These preliminary results suggest preferential routes by which HIV-1 may enter the male genital tract in female-to-male HIV sexual transmission.

# Sub article: WEAA0105: The HIV-1 envelope protein gp120 mimics MAdCAM and VCAM in binding to Integrin-α_4_β_7_

**Published:** 2012-10-22

**Authors:** F. Nawaz, C. Cicala, D. Van Ryk, D. Wei, M. Pascuccio, S. Shrestha, J. Knox, C. Schwing, J. Hiatt, J. Chang, K. Jelicic, A. Fauci, J. Arthos

**Affiliations:** 1 NIAID, National Institutes of Health, Bethesda, United States; 2 New York University School of Medicine, New York, United States

## Abstract

**Background:**

Our laboratory previously reported that HIV binds and signals through Integrin-α_4_β_7_, the gut homing receptor, on the surface of CD4^+^ T-cells. This interaction may be critical during the earliest events of HIV transmission, when the virus rapidly homes to the gut and a massive depletion of gut CD4^+^ T cells ensues. Understanding the precise manner by which the virus engages α_4_β_7_ may therefore provide insights into the earliest events in HIV infection and the design of novel therapeutics. Both its natural ligands (MAdCAM and VCAM) and gp120 bind α_4_β_7_ in a cation-dependent manner. In each case a divalent cation bound to the integrin coordinates an aspartic acid (Asp) residue in the ligand. We previously described a highly conserved Asp in the V2-loop of gp120 that mimics the natural ligands of α_4_β_7_, and plays a critical role in this interaction. However, MAdCAM and VCAM utilize a second Asp residue and a second coordinating cation to mediate recognition of both α_4_ and β_7_ chains in a complex way. Our data suggests that gp120 interactions with α_4_β_7_ are similarly complex.

**Methods:**

To identify critical residues in HIV-gp120 that mediate α_4_β_7_-reactivity we designed site-directed Asp mutants in recombinant gp120s, and measured the binding of each mutant to α_4_β_7_.

**Results:**

By this approach, we identified two sites that mediate gp120-α_4_β_7_ interactions. A single Asp mutant at either position reduced reactivity with α_4_β_7_ by~2-fold relative to the wildtype, while the double Asp mutants diminished α_4_β_7_ binding to near-undetectable levels.

**Conclusion:**

This observation reveals the discontinuous nature of the gp120-a_4__7_ epitope and suggests gp120 interactions with a_4__7_ closely mimic MAdCAM and VCAM. These results advance upon our previous understanding of the molecular basis of α_4_β_7_-gp120 interactions and lay the groundwork for further structural studies.

# Sub article: MOAA0203: HIV-1 peptide-specific NK cell responses in HIV seropositive and highly exposed seronegative men

**Published:** 2012-10-22

**Authors:** R. Fecek, R. Mailliard, C. Rinaldo

**Affiliations:** 1 University of Pittsburgh Graduate School of Public Health, Infectious Diseases and Microbiology, Pittsburgh, United States

## Abstract

**Background:**

Highly exposed seronegative (HESN) individuals have had repeated exposures to HIV-1 yet remain virus and antibody negative. We evaluated HIV-1 specific NK cell responses in men who have sex with men (MSM) defined as HESN based on high-risk sexual activities engaged in the early 1980's.

**Methods:**

Fresh, whole blood samples from HIV-1 seropositives on ART (HIVpos), HIV-1 seronegatives (HIVneg), and HESN subjects in the Multicenter AIDS Cohort Study were cultured with overlapping 15-mer peptide pools representing consensus sequences of HIV-1 Gag, Env, and Reg (Tat, Rev, Vif, Vpu, Vpr), or peptide diluent (Tiemessen, et al., JID 2010). The cultures were analyzed for CD3^-^CD56^+^ NK cell and CD3^+^CD8^+^ T cell responses by flow cytometry.

**Results:**

HIV-1 peptide-specific NK cell IFN-g and TNF-a responses were present in 19/23 (83%) HIVpos, 6/13 (46%) HESN and 4/21 (19%) HIVneg (P< 0.001 and P=0.07 compared to HIVpos and HESN, respectively). The IFN-g response magnitudes ranged from 1% to 20% of all NK cells: HIVpos=5.6±1.1%, HESN=1.5±0.7%, and HIVneg=0.47±0.1% (P< 0.001 and P=0.065 compared to HIVpos and HESN, respectively). HIVpos NK cells predominately responded to Env peptides, whereas HESN NK cells responded to both Env and Reg peptides. While both HIVpos and HESN demonstrated CD8^+^ T-cell responses to Env and Reg, only HESN had CD8^+^ T-cell IFN-g reactivity to Reg in association with NK cell responses.

**Conclusion:**

We show for the first time that contemporaneous blood samples from MSM defined as HESN exhibit relatively robust, innate NK cell immunity, and less common CD8^+^ T cell immunity, specific for HIV-1 proteins. These presumably long lasting, NK cell responses to Env and Reg peptides could be due to prior exposure to HIV-1, or to an inherited genetic resistance. In-depth assessment of these virus-specific NK cell responses could be important in designing effective HIV-1 therapeutics and vaccines.

# Sub article: WEPDA0102: Highly-exposed HIV seronegative persons (HESN) had higher levels of inflammatory and immune activation-associated serum biomarkers than lower risk HIV seroconverters (LRSC)

**Published:** 2012-10-22

**Authors:** O. Martinez-Maza, R. Detels, L. Magpantay, J. Phair, J.H. Bream, C.R. Rinaldo, L.P. Jacobson

**Affiliations:** 1 David Geffen School of Medicine at UCLA, UCLA AIDS Institute, Los Angeles, United States; 2 UCLA School of Public Health, Epidemiology, Los Angeles, United States; 3 David Geffen School of Medicine at UCLA, Obstetrics & Gynecology, Los Angeles, United States; 4 Northwestern University, Medicine - Infectious Diseases, Chicago, United States; 5 Johns Hopkins University Bloomberg School of Public Health, Molecular Microbiology and Immunology, Baltimore, United States; 6 University of Pittsburgh, Graduate School of Public Health, Infectious Diseases and Microbiology, Pittsburgh, United States; 7 Johns Hopkins University Bloomberg School of Public Health, EpidemiologyUnited States, Baltimore

## Abstract

**Background:**

Marked differences are seen in individuals’ susceptibility to HIV infection. Inflammation and immune activation are critical factors contributing to initial infection with HIV. The objective of this study was to define serum levels of cytokines and biomarkers of inflammation in participants in the Multicenter AIDS Cohort Study (MACS) who were at high risk for acquiring HIV infection but remained HIV seronegative (HESN), and in those at lower risk who did (LRSC), or did not (LRSN), subsequently undergo HIV seroconversion.

**Methods:**

Serum levels of cytokines, and biomarkers for inflammation were quantified using Luminex multiplexed assays, in 188 HESN, 125 LRSC, and 197 LRSN. LRSC were tested at a study visit preceding HIV seroconversion. HESN were defined as persistently seronegative participants who were multiply-exposed (>45 anal sexual partners in the 2.5 years prior to MACS visit 2). The LRSC and LRSN groups had<20 anal partners during this same period. CCR5D32 homozygotes were excluded from the HESN group. Age-adjusted left-censored generalized gamma regression models were used to compare levels and logistic regression models for detectability across groups.

**Results:**

HESN men demonstrated significantly lower age-adjusted serum levels of APO-A1 (P=0.036) and higher levels of sTNFR2 (P=0.022) than LRSC. Additionally, HESN men demonstrated significantly higher serum levels of sTNFR2 (P=0.014), sCD27 (P=0.035), sCD14 (P=0.014), and IL-8 (P< 0.004), and lower serum levels of CXCL13 (P=0.034) and IFNg (P=0.22), than LRSN. The likelihood of having detectable IL-2, IL-4, and IL-12 was higher in LRSN vs HESN, but detection of these biomarkers was similar between LRSC and HESN men.

**Conclusion:**

HESN men displayed lower age-adjusted serum levels of an anti-inflammatory molecule, APO-A1, and higher levels of a molecule associated with enhanced TNF responses (sTNF-R2) than did LRSC. These results are consistent with enhanced immune responsiveness and inflammation being associated with resistance to persistent infection with HIV.

# Sub article: WEAA0203: Target cell restriction may limit mother-to-child transmission of SIV in sooty mangabeys

**Published:** 2012-10-22

**Authors:** A. Chahroudi, D. Carnathan, P. Carnathan, G. Silvestri

**Affiliations:** 1 Emory University School of Medicine, Pediatrics, Atlanta, United States; 2 Yerkes National Primate Research Center, Atlanta, United States

## Abstract

**Background:**

Mother-to-infant transmission (MTIT) of HIV results in ~400,000 infected children each year, with a transmission rate of 35–40%. In contrast, nonhuman primate species that are naturally infected with SIV in the wild (“natural hosts”, including sooty mangabeys, SMs) rarely transmit SIV from mothers to infants. The mechanisms underlying this protection are unknown. In this study we tested the hypothesis that limited target cell availability protects SMs from MTIT.

**Methods:**

The availability of target cells (CD4+CCR5+ and CD4+Ki67+ T-cells) for SIV infection in seven uninfected SM infants was measured by flow cytometry among naïve and memory T-cell subsets obtained from tissues (lymph nodes, spleen, tonsil, and multiple sites along the gastrointestinal tract) at necropsy.

**Results:**

We found that, in infant SMs, the median percentage of CD4+Ki67+ T-cells ranged from 3.2%-10.3% depending on the anatomic site analyzed, with lower values found in CD95-CD28+ naïve CD4+ T-cells. In contrast, the CD95+CD28+CCR7+ central memory and CD95+CD28+CCR7- transitional memory CD4+ T-cells displayed higher median levels of Ki67 (10.4%-49.3%). The percentage of Ki67+CD95+CD28-CCR7- effector memory CD4+ T-cells tended to be between that of the naïve cells and other memory subsets. Despite this increased level of proliferation (compared to adult SMs), CCR5+T-cells comprised<10% (and, in most cases,<5%) of the CD4+ lymphocyte population at all sites and within all T cell subsets, thus revealing restricted expression of the SIV coreceptor in infant SMs.

**Conclusion:**

We have shown that MTIT is substantially less frequent in SIV-infected SMs than in HIV-infected humans. Here we demonstrate that while robust T-cell proliferation is present in infant SMs, SIV target cells (CD4+CCR5+ T-cells) are extremely limited in multiple tissues. This finding reveals an additional, previously unrecognized feature of the evolutionary adaptation to reduce the risk of MTIT in SIV-infected SMs.

# Sub article: TUAA0301: Pre-clinical evaluation of HIV replication inhibitors that target the HIV-integrase-LEDGF/p75 interaction

**Published:** 2012-10-22

**Authors:** F. Christ, C. Pickford, J. Demeulemeester, S. Shaw, B.A. Desimmie, C. Smith-Burchnell, S. Butler, M. Westby, Z. Debyser

**Affiliations:** 1 KULeuven, Laboratory for Molecular Virology and Gene Therapy, Leuven, Belgium; 2 Pfizer Inc, Sandwich, United Kingdom

## Abstract

**Background:**

Current HIV-1 integrase inhibitors target the catalytic activity, which is vital for sustained viral infection. Integrase mediates the critical step of proviral DNA integration. Efficient integration also requires a crucial cellular co-factor of HIV-1 integrase, LEDGF/p75, that tethers the viral DNA to the host chromatin. LEDGINs, small molecules designed to bind to the LEDGF/p75 interaction site on IN and to disrupt the interaction, have recently been shown to act as potent inhibitors of HIV replication in cell culture.

**Methods:**

We have now analyzed the detailed mode of action of LEDGINs, dissecting the allosteric nature of inhibition *in vitro* and analyzing phenotypically their activity in cell culture. Mechanistic studies and combination experiments shed light on potential synergy of LEDGINs with known integration inhibitors. Analysis of the inhibition of a broad spectrum of HIV strains allows predictions on combinations with other drugs such as entry, RT and protease inhibitors.

**Results:**

Biochemical evaluation of LEDGINs demonstrates in addition to a potent inhibition of the integrase-LEDGF/p75 interaction, a block of the catalytic integrase activities. This allosteric inhibition is promoted by the stabilization of the dimer-interface of IN upon LEDGIN binding most likely by affecting interaction with viral DNA. These properties of LEDGINs result in potent inhibition of HIV replication in both MT2 and PBMC cells. Moreover, LEDGINs are active across a broad range of HIV clades. LEDGINs retained full activity against a panel of viruses containing mutations that confer resistance to integrase strand transfer inhibitors. Combining LEDGINs with strand transfer inhibitors demonstrates a synergistic effect of these classes of integration inhibitors.

**Conclusion:**

The biochemical data together with the lack of cross resistance and the observed synergistic effects of LEDGINs in combination with strand transfer inhibitors support the potential for combined use of LEDGINs with strand transfer inhibitors in HIV therapy.

# Sub article: WEPDC0103: Development of a new intravaginal ring technology for the extended delivery of the microbicide tenofovir with and without the contraceptive levonorgestrel

**Published:** 2012-10-22

**Authors:** T. Johnson, M. Clark, J. Clark, N. Shelke, G. Doncel, D. Friend, P. Kiser

**Affiliations:** 1 University of Utah, Bioengineering, Salt Lake City, United States; 2 CONRAD, Eastern Virginia Medical School, Obstetrics & Gynecology, Arlington, United States; 3 CONRAD, Eastern Virginia Medical School, Obstetrics & Gynecology, Norfolk, United States; 4 University of Utah, Bioengineering Pharmaceutics and Pharmaceutical Chemistry, Salt Lake City, United States

## Abstract

**Background:**

Tenofovir (TFV) is the only microbicide antiretroviral that has shown clinical effectiveness when dosed pericoitally in a vaginal gel. There remains a need to improve TFV delivery by providing long-lasting, coitally-independent, effective drug levels. Intravaginal rings (IVRs) offer this capability; however TFV is hydrophilic and demonstrates inadequate delivery from conventional IVR technologies. Our objective was to develop a novel IVR technology capable of releasing ≥10 mg/d TFV for ≥90 days, alone or co-delivered with 10 or 20 µg/d of the contraceptive levonorgestrel (LNG).

**Methods:**

IVRs were designed using single (TFV-only) or dual (TFV-LNG) reservoir-type polyurethane (PU) segments. TFV segments comprised water-swellable PU tubing filled with a high density TFV paste. LNG segments comprising a LNG-loaded non-swellable PU core with a rate controlling membrane were cut to 1 and 2 cm lengths to obtain target release rates of 10 and 20 µg/d, respectively. In vitro release testing (IVRT) and 3-month pharmacokinetic (PK) studies in rabbits and sheep evaluated device performance.

**Results:**

IVRT revealed time-independent and tunable TFV and LNG release rates which were optimized to achieve our target 10 mg/d TFV and 10 or 20 µg/d LNG. In sheep, TFV and LNG release rates were estimated at ~12–17 mg/d and ~14–31 µg/d, respectively. TFV vaginal tissue and fluid levels were ~10^4^ and 10^6^ ng/g, respectively, similar to levels reported after clinical dosing of TFV 1% gel. In rabbits, LNG PK demonstrated 2-fold differences in plasma and cervical tissue concentrations between the two dose groups, as predicted by in vitro release.

**Conclusion:**

We developed a unique IVR technology that met our target product profile delivering a high flux of a hydrophilic antiretroviral (TFV) alone or with a low flux of a hydrophobic drug (LNG) in a controlled, time-independent manner. PK results are highly encouraging and warrant a Phase I clinical study. *[Picture of prototype TFV and TFV/LNG IVRs]*

**Figure 1:** **Picture of prototype TFV and TFV/LNG IVRs**.

# Sub article: TUAA0302: Towards HIV eradication: excision of HIV-1 proviral DNA by Tre-recombinase in HIV-positive humanized mice

**Published:** 2012-10-22

**Authors:** H. Hofmann-Sieber, I. Hauber, J. Chemnitz, A. Grundhoff, J. Chusainow, A. Schambach, C. Baum, P. Ziegler, M. Manz, F. Buchholz, J. Hauber

**Affiliations:** 1 Heinrich Pette Institute-Leibniz Institute for Experimental Virology, Hamburg, Germany; 2 University of Technology Dresden, University Hospital and Medical Faculty Carl Gustav Carus, Department of Medical Systems Biology, Dresden, Germany; 3 Hannover Medical School, Institute of Experimental Hematology, Hannover, Germany; 4 Institute for Research in Biomedicine, Bellinzona, Switzerland; 5 University Hospital Zurich, Zurich, Switzerland

## Abstract

**Background:**

HIV-1 integrates into the host chromosome and persists as a provirus flanked by long terminal repeats (LTR). To date, treatment regimens primarily target the virus enzymes or virus entry, but not the integrated provirus. Therefore, HAART requires lifelong treatment which is frequently accompanied by the occurrence of substantial side effects and/or the development of drug-resistant viruses. Previously, we engineered a LTR-specific recombinase (Tre-recombinase) that effectively excises integrated HIV-1 proviral DNA from infected human cell cultures, suggesting that customized enzymes might someday help to eradicate HIV-1 from the body. Therefore, we here analyzed the potential of Tre-recombinase to reverse HIV-1 infection in vivo.

**Methods:**

We constructed an advanced lentiviral self-inactivating (SIN) vector that expresses Tre-recombinase conditionally in HIV-infected cells. We monitored Tre functionality and potential Tre-related cytopathic effects over time in tissue cultures. Moreover, the effect of Tre activity on HIV-1 infection was investigated in humanized mice.

**Results:**

It is shown that Tre-recombinase is efficiently delivered into cells and accurately excises HIV-1 proviral DNA from chromosomal integration sites. Apparently, prolonged overexpression of Tre-recombinase does not induce undesired cytopathic effects in the transduced cells. Finally, we demonstrate pronounced antiviral activity of Tre-recombinase in HIV-1 infected Rag2^−/−^?c^−/−^ mice, which were either engrafted with Tre-transduced human CD4^+^ T cells or with Tre-transduced human CD34^+^ hematopoietic stem cells (HSC).

**Conclusion:**

The presented data suggest that Tre-recombinase may be a valuable component of future antiretroviral therapies of the post HAART era that aim at virus eradication, thereby providing a cure for AIDS.

# Sub article: TUAA0303: *In vivo* suppression of HIV by antigen specific T cells derived from engineered hematopoietic stem cells

**Published:** 2012-10-22

**Authors:** S. Kitchen, B. Levin, G. Bristol, V. Rezek, S. Kim, C. Aguilera-Sandoval, A. Balamurugan, O. Yang, J. Zack

**Affiliations:** 1 David Geffen School of Medicine at UCLA, UCLA AIDS Institute, Los Angeles, United States

## Abstract

**Background:**

In HIV infection, the HIV-specific cytotoxic T lymphocyte (CTL) response is a critical component in controlling viral replication that ultimately fails in its ability to eradicate the virus from the body. Our primary aim is the development of a way to enhance the HIV-specific CTL response to allow long-term viral suppression or viral clearance.

**Methods:**

In our approach, we sought to genetically manipulate human hematopoietic stem cells (HSCs) such that they differentiate into mature CTLs that will kill HIV infected cells. To perform this, we utilized molecularly cloned HIV-specific T cell receptors (TCRs) derived from CD8+ T cells. These TCRs were used to genetically transduce HSCs that were introduced into a humanized mouse and were allowed to differentiate into mature human CD8+ CTLs. Mice expressing the transgenic HIV-specific TCR and, separately, control mice were then infected with HIV-1 and functional cellular responses, viral suppression, and viral and T cell dynamics were assessed.

**Results:**

We found that genetic modification of human HSCs with a cloned TCR allows proper differentiation of the cells to occur *in vivo* and these cells migrate to multiple anatomic sites, mimicking what is seen in humans. We observed that the genetically modified HIV-specific CTLs form a functional antiviral response *in vivo* that results in the significant suppression of HIV replication in multiple organs. In addition, we found significant correlations between the levels of reconstitution with cells bearing the HIV-specific TCR, antigen-driven T cell expansion, and the control of viral replication.

**Conclusion:**

We have developed a system to closely characterize the engineering of antiviral immunity and HIV-specific CTL responses. Our results strongly suggest that stem cell based gene therapy may be a feasible approach in the treatment of chronic viral infections and provide a foundation towards the development of this type of strategy.

# Sub article: THPDA0202: Hematopoietic stem cell gene therapy targeting HIV-1 based on lentiviral CCR5Δ32 and multiple microRNAs

**Published:** 2012-10-22

**Authors:** L.-J. Chang, G. Alkhatib, L.-M. Huang, Y. Chen, Y. Yeh

**Affiliations:** 1 University of Florida, Molecular Genetics and Microbiology, Gainesville, United States; 2 Texas Tech University Health Sciences Center, Biomedical Sciences, El Paso, United States; 3 National Taiwan University, Pediatrics, Taipei, Taiwan, Province of China

## Abstract

**Background:**

Treatment of HIV infection by antiretroviral therapy is effective but costly and often associated with numerous side effects. The key to a permanent treatment to chronic HIV infections is to elicit potent host resistance to viral infection and to restore immune functions. The prolonged incubation period of HIV-1 provides a good opportunity for applying non-conventional interventions such as gene therapy. For HIV gene therapy to be effective, the combination of an efficient gene transfer vector and a powerful anti-HIV strategy is necessary.

**Methods:**

HIV resistance will be established in patients′ hematopoietic stem cells (HSCs) by lentivector (LV) transduction of (i) a microRNA to block endogenous CCR5 expression, (ii) a sequence-modified CCR5Δ32 gene to interfere with the function of native CCR5 and CXCR4 and (iii) effective multiple anti-HIV shRNAs to target viral RNAs.

**Results:**

We generated LVs encoding the native and a codon-optimized CCR5Δ32 gene. Ectopic expression of CCR5Δ32 in HOS-R5 and Magi-R5 cells established protection against R5-HIV-1 infection. Unexpectedly, we observed severe cytotoxicity in HOS-R5 cells and primary CD4 T cells when CCR5Δ32 was expressed. In a second approach, we generated a LV expressing an H1-promoter driven CCR5 miRNA and demonstrated marked protection against R5-HIV-1 infection. In a third approach, we generated a novel LV expressing three miRNA intronic cassettes (miR155-19a-30a) targeting HIV-1 pol, int and vpu, respectively, and demonstrated marked protection against HIV-1 infection. LV transduction of adult CD34+ HSCs had no adverse effect on hemopoiesis for dendritic cell development but T cell development appeared to be impaired based on an *in vitro* assay.

**Conclusion:**

We conclude that ectopic expression of CCR5Δ32 in adult CD34+ HSCs using a constitutive expression promoter is cytotoxic because the CCR5Δ32 transgene can activate uncontrollable intracellular T cell signaling. However, miRNAs targeting endogenous CCR5 and multiple HIV sequences is highly effective against HIV infection without cytotoxicity.

# Sub article: MOLBA05: Stabilized exposure of conserved epitopes by structure guided insertion of disulfide bonds in HIV envelope glycoprotein

**Published:** 2012-10-22

**Authors:** A. Dey, A. Kassa, A. Nandi, P. Sarkar, Y. Sun, K. Hartog, C. Labranche, D. Montefiori, I. Srivastava, A. Carfi, S. Barnett

**Affiliations:** 1 Novartis Vaccines & Diagnostics Inc., Cambridge, United States; 2 Novartis Vaccines & Diagnostics Inc., Emeryville, United States; 3 Duke University Medical Center, Durham, United States; 4 Vaccine Research Center / NIAID/NIH, Vaccine Production Program Laboratory / VRC/NIAID, Bethesda, United States

## Abstract

**Background:**

The transition from native to receptor-bound and eventually to the fusion-competent conformation of HIV-1 envelope glycoprotein (Env) Env requires a tightly choreographed interaction among highly conserved structures within the viral envelope glycoprotein. Recent structural information has revealed that these conformational transitions are regulated by three conserved but highly mobile layers stacked between the receptor-binding domain (gp120) and the fusion arm (gp41) of Env. We hypothesized that limiting the mobility of these layers by artificially insertion of covalent bonds will capture Env in a conformation where conserved sites are stably exposed.

**Methods:**

We scanned residues that lie at the interface of gp120 layers (layers 1, 2 and 3) and identified targets that are predicted to form disulfide bonds if substituted with paired cysteines. We substituted a pair of residues between layers 1 and 2 with cysteine & expressed and purified the disulfide-stabilized gp120 (and gp140) antigens and analyzed them using Surface Plasmon Resonance (SPR) assay. Following in vitro analysis, we immunized rabbits with both the wild-type and disulfide-stabilized gp120 (and gp140) antigens, adjuvanted with Carbopol+MF59, and evaluated serum antibodies for binding, neutralization and epitope-specificity.

**Results:**

A single disulfide bond inserted between the highly conserved layers 1 and 2 led to enhanced stability of the receptor bound conformation of gp120. This was revealed by lower dissociation constant (Kd) of the mutant gp120 binding to 17b antibody, which recognized a conserved CD4 induced (CD4i)-epitope on gp120. Upon immunization in rabbits, the disulfide-stabilized gp120 (and gp140) antigens, in comparison to wild-type antigens, elicited higher level of antibodies directed to CD4-binding site and CD4i-site.

**Conclusion:**

We demonstrate that structure guided stabilization of inter-layer interactions within Env can be used to improve and stabilize the exposure of conserved epitopes on the antigen and elicit improved antibody response, with the aid of a potent adjuvant, upon immunization.

# Sub article: TUPDA0103: Novel DNA vaccine candidates that mimic the neutralization-competent structure of the MPER of HIV-1 gp41

**Published:** 2012-10-22

**Authors:** N. Gulzar, M. Montero, K.-A. Klaric, C. Lepik, J. Donald, S. Tsai, S. Wu, S. Wang, W. Degrado, S. Lu, J.K. Scott

**Affiliations:** 1 Simon Fraser University, Burnaby, Canada; 2 University of Pennsylvania, Philadelphia, United States; 3 University of Massachusetts Medical School, Worcester, United States; 4 University of California-San Francisco, San Francisco, United States; 5 Simon Fraser University, MBB/FHS, Burnaby, Canada

## Abstract

**Background:**

The limited success of vaccines targeting the MPER of HIV-1 gp41, we hypothesize, may in part reflect the difficulty of mimicking its neutralization-competent structure (NCS). We have developed DNA-vaccine candidates meant to emulate the NCS of the MPER, and report on their ability to elicit MPER-specific, neutralizing (Nt) antibodies (Abs).

**Methods:**

DNA vaccines encoding various gp41 ectodomain fragments, and the transmembrane region (TM) of either the platelet-derived growth factor receptor (PGDFR), or that of gp41 were engineered, transiently expressed in COS-7 cells, and tested for antigenicity. Rabbits were immunized with plasmid DNA of select candidates; sera were collected and tested for MPER reactivity.

**Results:**

Work with the protein products of these vaccines has shown they mimic the NCS of the MPER by several criteria, including the ability to be bound tightly by well-characterized Nt MAbs, but weakly by their non-neutralizing mutant-MAb counterparts. Immunizations with plasmids expressing the MPER tethered to the PDGFR-TM elicited MPER-specific Abs that targeted the epitope of the 2F5 NtAb. Immunization with DNA vaccines encoding the MPER and gp41 TM, elicited low-titre Abs that cross-reacted weakly with the MPER, and strongly with regions outside the MPER. Both sets of immunizations failed to produce Abs that cross-reacted with the 4E10 epitope, or neutralized pseudoviruses bearing HIV-1 Env. We found that the presence of the PGDFR-TM significantly reduced MPER-binding to 4E10 MAb, but not by 2F5. Putative models suggest that in the PDGFR-TM fusions, the 4E10 epitope faces into the lipid bilayer, thereby altering its exposure.

**Conclusion:**

Our work reveals key structural features involved in promoting the NCS of the MPER. While the gp41 TM is vital in properly exposing neutralizing epitopes on the MPER, it was also found to elicit Abs against sites outside the MPER. Current work is focused on engineering the gp41 TM to optimally expose MPER epitopes.

# Sub article: WEAA0102: A novel HIV vaccine approach: targeting the protease cleavage sites of HIV-1

**Published:** 2012-10-22

**Authors:** M. Luo, D. Tang, R. Capina, X.-Y. Yuan, C. Prego, J.C. Pinto, M. Alonso, C. Barry, R. Pilon, C. Daniuk, J. Tuff, S. Pillet, D. La, T. Bielawny, C. Czarnecki, P. Lacap, H. Peters, G. Wong, M. Kimani, C. Wachihi, J. Kimani, T. Ball, P. Sandstrom, G. Kobinger, F. Plummer

**Affiliations:** 1 National Microbiology Laboratory, HIV and Human Genetics, Winnipeg, Canada; 2 University of Manitoba, Medical Microbiology, Winnipeg, Canada; 3 University of Santiago de Compostela, Pharmacy and Pharmaceutical Technology, Santiago, Spain; 4 University of Nairobi, Nairobi, Kenya

## Abstract

**Background:**

The classical vaccine approach for combating other viruses has failed so far in dealing with HIV-1, a virus infecting a key component of immune system and with greater diversity and rapid mutation. New approaches are needed to develop a preventative vaccine.

The protease of HIV-1 is a small 99-amino acid aspartic enzyme mediating the cleavage of Gag, Gag-Pol and Nef precursor polyproteins. The process is highly specific, temporally regulated and essential for the production of infectious virions. A total of 12 proteolytic reactions are required to generate a viable virion. Therefore, a vaccine targeting the 12 protease cleavage sites(PCS) could be effective. The PCS of HIV-1 are highly conserved, direct immune responses against these sites would yield several advantages. First, the immune response could destroy the virus before its establishment in the host. Second, it could force the virus to accumulate mutations eliminating the normal function of the HIV protease. Third, restricting the immune responses to these sites can avoid distracting immune responses that often generate unwanted inflammatory responses, induce excess immune activation, and attract more targets for HIV-1 infection, establishment and spread.

**Methods:**

We conducted a pilot study to investigate the feasibility and effectiveness of this approach. The recombinant VSV-peptides were used to immunize cynomolgus macaques and nanopackaged peptides were used to boost the immune response to the 12 PCS of SIVmac239. The controls and immunized macaques were repeatedly challenged intrarectally with an increased dosage of SIVmac239.

**Results:**

Antibody and T cell responses to the 12 PCS can protect macaques against higher dosage of SIVmac239 challenge (p=0.0005, R=0.8005) and the vaccine group maintains significantly higher CD4+ counts (p=0.0002) than the controls weeks after being infected. Population coverage analysis showed that this approach can be applied to >95% populations in the world.

**Conclusion:**

Targeting 12 PCS of HIV-1 is a viable approach.

# Sub article: WEAA0101: Characterization of early gene expression profile in the blood of macaques immunized a Thai trail-like vaccine

**Published:** 2012-10-22

**Authors:** M. Vaccari, M. Cameron, N. Liyanage, P. Pegu, S. Gordon, M. Doster, R.-P. Sekaly, G. Franchini

**Affiliations:** 1 National Institute of Health, Bethesda, United States; 2 VGTI-Florida, Port St Lucie, United States

## Abstract

**Background:**

A strategy combining Canarypox based vaccine, ALVAC-HIV with gp120 protein has resulted in limited but significant protection from HIV infection in The RV144 vaccine efficacy trial. We have previously tested a similar strategy in the non-human primate model of HIV infection obtaining a similar efficacy to what observed in humans.

**Methods:**

To study the contribution of innate immune responses as correlate of protection, we have performed microarray analysis in the blood collected from 6 macaques at 16, 24 48 and 72 hours after the first two immunizations with ALVAC (V1 and V2 respectively) and the 3rd immunization with ALVAC/gp120 protein (V3).

**Results:**

Our results show that 1) 24h after the 1st immunization with ALVAC-SIV (V1) genes with antiviral activity were up regulated (MX1, HERC-5, CD79b), but interestingly inflammatory genes where down regulated (IL1, IL18RAP, IFNR1), suggesting a reciprocal regulation of genes for IFN type I and II; 2) similar patterns of gene expression where observed earlier, at 16h from V2, suggesting the presence of "memory -innate" anti viral responses; 3) the 3rd boost with ALVAC, given simultaneously to the gp120 protein adjuvanted in Alum (V3), resulted in significant changes in the gene expression profile when compared to the first two vaccinations. At 24h from this immunization there were a far less number of IFN-related genes that were significantly up regulated (V3=3) when compared to the first and second immunization (V1= 17; V2=27), and the IFN-responses still up regulated were associated with NK cells, B cell- (CD79B) and T cell- (PKC, CD28 TCR) responses.

**Conclusion:**

These results suggest that each component of vaccination could have contributed to the protection from infection underscored the ALVA/gp120 strategy. Understanding how to induce different types of immune responses that are protective for HIV may be relevant for the generation of more effective vaccine strategies.

# Sub article: THPDA0101: Peripheral blood monocytes contribute to the immune reconstitution inflammatory syndrome (IRIS): is the complement system emerging to the spotlights?

**Published:** 2012-10-22

**Authors:** H. Tran Thi Thanh, R. Van den Bergh, L. Kestens, M. Massinga-Loembe, R. Colebunders, P. De Baetselier, G. Raes, TB-IRIS Study Group

**Affiliations:** 1 Vrije Universiteit Brussel, Cellular and Molecular Immunology Unit, Brussels, Belgium; 2 Myeloid Cell Immunology Laboratory, VIB, Brussels, Belgium; 3 Institute of Tropical Medicine, Antwerpen, Belgium

## Abstract

**Background:**

One of the most frequent complications associated with antiretroviral therapy (ART) in HIV-tuberculosis (TB) co-infected patients is the Immune Reconstitution Inflammatory Syndrome (IRIS). While monocytes/macrophages play a major role in both HIV- and TB-infection individually, the putative contribution of monocytes to the development of TB-IRIS remains uncharacterized. We therefore applied a parallel approach of genome-wide microarray analysis and focused gene expression profiling in monocytes from Ugandan HIV-TB co-infected patients before and shortly after ART initiation, to investigate the possible functional contribution of monocytes to the development of IRIS.

**Methods:**

Monocyte gene expression of TB-IRIS patients and non-TB-IRIS patients was analyzed by Affymetrix GeneChip^®^ Human Gene 1.0 ST Arrays and was confirmed using the nCounter system; datasets were analyzed for overrepresented pathways using Ingenuity Pathway Analysis. Expression levels of and enzymatic activities of proteins of interest were characterized in the isolated monocyte fractions and in the plasma of IRIS patients.

**Results:**

Pathway analysis indicated that the complement system was significantly modulated in monocytes of TB-IRIS patients, both before initiation of therapy (baseline) and after two weeks of therapy. At baseline, expression of both C1q and C1-inhibitor was higher in TB-IRIS patients. After two weeks, the C1q mRNA levels in the majority of TB-IRIS patients increased, whereas C1-inhibitor mRNA levels decreased pronouncedly. Additionally, the inhibitory activity of C1-inhibitor was significantly higher in TB-IRIS patients compared with non-TB-IRIS patients at baseline but reduced to the level of C1-inhibitor activity in non-TB-IRIS patients at week 2.

**Conclusion:**

For the first time, we provide evidence that monocytes at least partially contribute to the development of TB-IRIS, probably through the complement system response. An intriguing possibility is that the relative balance between C1q and C1-inhibitor may be affecting the inflammatory function of C1q in the complement cascade.

# Sub article: THPDA0102: Cytokine storm in leishmania/HIV-1 co-infected patients can be caused by LPS and leishmania infection

**Published:** 2012-10-22

**Authors:** J. Santos-Oliveira, C. Giacoia-Gripp, E. Regis, P. Alexandrino-Oliveira, V. Amato, J.A. Lindoso, H. Goto, J. Guerra, M. Mattos, M. Oliveira-Neto, B. Grinzstejn, M. Morgado, A. Da-Cruz

**Affiliations:** 1 Oswaldo Cruz Foundation/ FIOCRUZ, Rio de Janeiro, Brazil; 2 Federal University of Mato Grosso do Sul, Rio de Janeiro, Brazil; 3 University of Sao Paulo (USP), Sao Paulo, Brazil; 4 Tropical Medicine Foundation, Manaus, Brazil

## Abstract

**Background:**

We have shown that leishmaniasis contributes to heightened T-cell activation in HIV-1/Visceral leishmaniasis (AVL) patients. Now we investigate whether lipopolysaccharide (LPS) has a crucial role in the pathogenesis of this co-infection.

**Methods:**

10 healthy volunteers, 17 AVL/HIV-1 and 16 HIV-1/AIDS patients were recruited. CD4^+^T counts and CD8^+^T cells expressing CD38 were analyzed by flow cytometry. LPS and sCD14 levels were measured by enzymatic assays. Plasmatic pro-inflammatory cytokines (IL-1/IL-6/IL-8/IL-17/IFN-γ/TNF-a) were assessed by multiplex analysis. The macrophage migration inhibition factor (MIF) and intestinal fatty acid binding protein (IFABP) were quantified by ELISA. Mann-Whitney and Spearman correlation test were employed for statistical analysis. Multivariate linear regression was used to determine influence of intervenient factors over T-cell activation.

**Results:**

AVL/HIV-1 patients in leishmaniasis remission presented equal CD4^+^T counts or T-cell activation levels in comparison to patients in the active phase of leishmaniaisis. Higher levels of CD8^+^/CD38^+^ were seen independently of leishmaniasis clinical phase when compared to HIV/AIDS cases (*p*< 0.05). Viral load levels had no influence in CD8^+^/CD38^+^ levels. Co-infected and HIV^+^ patients presented similar LPS and IFABP levels, but higher than healthy donors (*p*< 0.001). Pro-inflammatory cytokines levels, but not MIF were significantly augmented in co-infected cases. LPS levels were positively correlated with MIF (r=0.40;*p*< 0.05). We found positive correlation between LPS levels and CD38 on CD8^+^ T lymphocytes(p<0.001), independently of CD4^+^ T counts, HIV viremia, sCD14, MIF and IFAB levels. Leishmania infection was also positively correlated with activation levels(*p*< 0.001). We also observed that LPS and Leishmania infection were positively correlated with IL-6(p< 0.05) and IL-8(p< 0.01) levels.

**Conclusion:**

*Leishmania*/HIV-1 patients had an exacerbated pro-inflammatory cytokine response. In addition, LPS contributes to higher T-cell activation. In conclusion, *Leishmania* infection along with LPS levels may contribute to cytokine storm, which in turn may increase T-cell activation, worsening the condition of patients harboring both pathogens.

# Sub article: THPDA0104: HIV-facilitated paracellular penetration of HPV into mucosal epithelium

**Published:** 2012-10-22

**Authors:** S. Tugizov, R. Herrera, P. Veluppillai, D. Greenspan, J. Palefsky

**Affiliations:** 1 University of California at San Francisco, Medicine, San Francisco, United States; 2 University of California at San Francisco, Orofacial SciencesSan Francisco, United States

## Abstract

**Background:**

The incidence of HPV-associated lesions is higher in HIV-infected than in HIV-uninfected individuals. Oral and anogenital mucosal epithelia of HIV/AIDS-positive individuals contain infiltrating HIV-infected immune cells that express viral tat and gp120, and proinflammatory cytokines TNF-a and IFN-g. These proteins may disrupt tight junctions (TJ) of mucosal epithelium, facilitating HPV penetration. Our aims were to investigate how HIV-associated disruption of mucosal epithelium promotes HPV infection.

**Methods:**

Polarized oral and cervical epithelial cells and tissue explants from HIV-uninfected individuals were treated with recombinant HIV-1 tat, gp120, TNF-a and IFN-g independently and together. The cells and tissue explants were exposed to HPV 16 pseudovirions labeled with fluorescent dyes. Paracellular HPV penetration through disrupted epithelium was evaluated by confocal microscopy.

**Results:**

Treatment of oral and cervical epithelial cells with tat, gp120, TNF-a, or IFN-g independently for 24 h did not induce significant disruption of TJ but the combination of all 4 proteins caused disruption of TJ in about 90% of cells. Prolonged treatment of cells with these proteins independently for 5 days also induced substantial disruption of TJ. Epithelial disruption mediated by these proteins facilitated paracellular PsV passage through polarized cells-30-45% of apically applied virions were detected in the basolateral compartment. Treatment of oral epithelial explants with HIV tat, gp120, TNF-a, and IFN-g led to disruption of epithelial TJ with paracellular HPV penetration into epithelium and entry of HPV into basal cells.

**Conclusion:**

Our data indicate that HIV tat, gp120, TNF-a, and/or IFN-g in the epithelial microenvironment disrupt epithelial TJ in a time-dependent manner. Alone or together, they potentiate HPV penetration into basal epithelial cells where HPV infection is initiated. Interference with the effects of these proteins may be useful to reduce the risk of HPV infection when applied topically to at-risk genital mucosal epithelium prior to sexual exposure.

# Sub article: THPDA0105: Prevalence and genotypic variability of TT virus are extremely high in HIV-1-infected adults in the Lampang HIV-1 cohort in northern Thailand

**Published:** 2012-10-22

**Authors:** H. Thaisri, M. Moriuchi, N. Tsuchiya, A. Rojanawiwat, P. Pathipvanich, P. Sawanpanyalert, K. Ariyoshi, H. Moriuchi

**Affiliations:** 1 National Institute of Health, Bangkok, Thailand; 2 Nagasaki University Graduate School of Biomedical SciencesDepartment of Molecular Microbiology and Immunology, Nagasaki, Japan; 3 Nagasaki University, Institute for Tropical Medicine, Nagasaki, Japan; 4 Lampang Hospital, Lampang, Thailand

## Abstract

**Background:**

TT virus is genetically variable and widespread among the general population without apparent pathological effects. It has been detected in the peripheral blood and a variety of body fluids such as semen and cervical fluids. Studies from North America and Europe have reported that TTV infection is more prevalent in HIV-1-infected individuals than in healthy control, but its prognostic significance has been controversial. No study has been reported for TTV co-infection in HIV-1-infected Asian people. This study was aimed to demonstrate prevalence, genotypic variability and prognostic significance of TTV coinfection in northern Thailand HIV cohort.

**Methods:**

A total of 756 HIV-1-infected adults were enrolled in the Lampang HIV cohort in northern Thailand, and their blood samples were collected. HIV-1 plasma viral loads and CD4 counts were measured at enrollment, and their clinical courses had been monitored. 40 healthy Japanese adults were also tested as controls. DNA of 5 TTV genogroups (G1 to G5) was detected by PCR using genogroup-specific primer pairs.

**Results:**

753 (99.9%) of 754 HIV-1-infected adults were infected with any genogroup of TTV: G1 (700/754, 93%), G2 (0/754, 0%), G3a (740/754, 98%), G3b (732/754, 97%), G4 (601/754, 80%) and G5 (562/754, 75%). On the other hand, 38 (95%) of 40 healthy Japanese adults were infected with any TTV genogroup: G1 (63%), G2 (3%), G3a (70%), G3b (68%), G4 (63%) and G5 (20%). Infection with more than one genogroup is more common in HIV-1-infected adults (99%) than in controls (68%). 62% HIV-1-infected and only 13% healthy adults were infected with all of G1, G3a, G3b, G4 and G5.

**Conclusion:**

TTV infection is highly prevalent in both HIV-1-infected and uninfected Asian adults; however, mixed genogroups were much more common in the former. Correlations between certain TTV genogroup or TTV loads and CD4 counts or HIV-1 load will be determined.

# Sub article: TUAA0105: A universal nanoparticle cell secretion capture assay for the study of HIV-1-positive tissues

**Published:** 2012-10-22

**Authors:** W. Fitzgerald, J.-C. Grivel

**Affiliations:** 1 NIH, NICHD, Bethesda, United States

## Abstract

**Background:**

Secreted proteins play an important role in intercellular interactions, especially between cells of the immune system. Infection of human lymphoid tissues by HIV-1 may alter secretion patterns. Here, we describe a novel, easy, inexpensive, and versatile method, which allows the identification and isolation of a living cell actually secreting any protein of interest.

**Methods:**

We coupled carboxylated magnetic iron oxyde nanoparticles (IONPs) or quantum dots to polyclonal goat anti-mouse IgG(H+L) antibodies (GAM) and used them as a platform to bind a cell-specific antibody, conferring cell targeting, and an antibody specific for a secreted protein. Purified complexed IONPs form an affinity matrix on the cell surface and capture the cell-secreted product, which can then be detected on the surface of the secreting cell by another secreted-protein-specific labeled antibody.

**Results:**

GAM-IONPs complexed with anti-CD45 antibody and either anti-IL-2 or anti-IFNgamma. This capture assay was as efficient as a commercial assay and intracellular cytokine staining in identifying cells that secrete IL-2 and IFNg. Respectively, these assays detected IL-2 secretion on 16.9±4%, 14.7±1.8% and 16.3±1.4% of T cells (N=6, P<0.88) in activated PBMC cultures. Similarly, IFNg secretion was detected equally by these three assays. Quantum dots can be used in place of IONPs and allowed similar detection of cytokines as well as fluorescent targeting of cells. The capture assay also detected the secretion of MIP-1a, MIP-1b and RANTES for which no commercial assays are available, and levels were comparable to intracellular staining. This method is amenable to multiplexing and several cytokines may be detected on the same cells.

**Conclusion:**

We have developed a versatile secretion capture assay, which allows the detection of any secreted protein of interest, and does not compromise cell viability. This method is useful in understanding modifications to cytokine secretion induced by viruses such as HIV-1.

# Sub article: TUPDE0203: Evaluation of the use of dried blood spots for viral load monitoring in resource-limited settings in Zimbabwe

**Published:** 2012-10-22

**Authors:** S. Ngwende, B. Mudenge, D. Madzimure, G. Mudenge

**Affiliations:** 1 National Microbiology Reference Laboratory, Ministry of Health and Child Welfare, Harare, Zimbabwe; 2 Flow Cytometry Laboratory, Harare, Zimbabwe

## Abstract

**Background:**

Monitoring of antiretroviral treatment (ART) with human immunodeficiency virus (HIV) viral loads, is rarely available in resource-limited settings because of the high costs, stringent requirements for storage and transport of plasma. Monitoring of antiretroviral therapy (ART) with HIV-1 viral load assays to determine virological treatment failure and to decide when to change to second line therapy is highly recommended. Requirements for -80 degrees Celsius freezers for sample storage prohibit implementation of this level of care in resource poor settings. Dried blood spots (DBS) can be used as an alternative to plasma, but the use of DBS for HIV-1 assays has not been assessed in Zimbabwe. This study investigates the performance of DBS for HIV viral load monitoring of patients on ART in Zimbabwe.

**Methods:**

Parallel forty eight (48) plasma samples and 48 DBS samples were used in this study. They were selected from samples stored at Flow Cytometry Laboratory, Zimbabwe, collected from patients with ART failure and who were being monitored for HIV drug resistance. Viral load assays were performed on 48 samples using plasma and dried blood spots methods. Plasma was separated and frozen at -80 degrees Celsius and DBS were kept at room temperature for 30days.

**Results:**

The correlation between plasma and DBS viral load was high (R2=0.75). Mean difference was 0.05 log10 copies/mL (SD 0.58), and only 8 samples showed >1 log10 difference. Sensitivity and specificity of DBS to detect virological failure (plasma viral load >400 copies/mL) was 82.5 and 93.1%, respectively.

**Conclusion:**

There was a very good correlation between DBS stored at room temperature for 30 days and plasma viral load assays. The evaluation shows that DBS can be a feasible and reliable method for virological monitoring of patients on ARVs in rural areas with transport facilities for referring samples to a central laboratory.

Abstract Coding GuideExample: **MOAA01**=(Weekday) **MO** – (Session type) **AA** – (Session order) **01**

Weekdays: SU (Sunday), MO (Monday), TU (Tuesday), WE (Wednesday), TH (Thursday), FR (Friday)

Session types: oral abstract sessions – AA (Track A), AB (Track B), AC (Track C), AD (Track D), AE (Track E), AX (Cross-Track), LBA (Late Breaker Track A), LBB (Late Breaker Track B), LBC (Late Breaker Track C), LBD (Late Breaker Track D), LBE (Late Breaker Track E), LBX (Late Breaker Cross-Track); oral poster discussions sessions – PDA (Track A), PDB (Track B), PDC (Track C), PDD (Track D), PDE (Track E) PDX (Cross-Track)

Session order: 01, 02, 03, 04, etc.
